# Activation of the Canonical Bone Morphogenetic Protein (BMP) Pathway during Lung Morphogenesis and Adult Lung Tissue Repair

**DOI:** 10.1371/journal.pone.0041460

**Published:** 2012-08-20

**Authors:** Alexandros Sountoulidis, Athanasios Stavropoulos, Stavros Giaglis, Eirini Apostolou, Rui Monteiro, Susana M. Chuva de Sousa Lopes, Huaiyong Chen, Barry R. Stripp, Christine Mummery, Evangelos Andreakos, Paschalis Sideras

**Affiliations:** 1 Biomedical Research Foundation of Academy of Athens, Centre for Immunology & Transplantations, Athens, Greece; 2 Dept Anatomy and Embryology, Leiden University Medical Centre, Leiden, The Netherlands; 3 Division of Pulmonary, Allergy and Critical Care, Duke University Medical Centre, Durham, North Carolina, United States of America; University of Pennsylvania School of Medicine, United States of America

## Abstract

Signaling by Bone Morphogenetic Proteins (BMP) has been implicated in early lung development, adult lung homeostasis and tissue-injury repair. However, the precise mechanism of action and the spatio-temporal pattern of BMP-signaling during these processes remains inadequately described. To address this, we have utilized a transgenic line harboring a BMP-responsive eGFP-reporter allele (BRE-eGFP) to construct the first detailed spatiotemporal map of canonical BMP-pathway activation during lung development, homeostasis and adult-lung injury repair. We demonstrate that during the pseudoglandular stage, when branching morphogenesis progresses in the developing lung, canonical BMP-pathway is active mainly in the vascular network and the sub-epithelial smooth muscle layer of the proximal airways. Activation of the BMP-pathway becomes evident in epithelial compartments only after embryonic day (E) 14.5 primarily in cells negative for epithelial-lineage markers, located in the proximal portion of the airway-tree, clusters adjacent to neuro-epithelial-bodies (NEBs) and in a substantial portion of alveolar epithelial cells. The pathway becomes activated in isolated E12.5 mesenchyme-free distal epithelial buds cultured in Matrigel suggesting that absence of reporter activity in these regions stems from a dynamic cross-talk between endoderm and mesenchyme. Epithelial cells with activated BMP-pathway are enriched in progenitors capable of forming colonies in three-dimensional Matrigel cultures.

As lung morphogenesis approaches completion, eGFP-expression declines and in adult lung its expression is barely detectable. However, upon tissue-injury, either with naphthalene or bleomycin, the canonical BMP-pathways is re-activated, in bronchial or alveolar epithelial cells respectively, in a manner reminiscent to early lung development and in tissue areas where reparatory progenitor cells reside. Our studies illustrate the dynamic activation of canonical BMP-pathway during lung development and adult lung tissue-repair and highlight its involvement in two important processes, namely, the early development of the pulmonary vasculature and the management of epithelial progenitor pools both during lung development and repair of adult lung tissue-injury.

## Introduction

Mammalian lungs are designed to optimize exposure of blood to oxygen. To achieve this, two intertwined and highly branched tree-like tubular systems, one conducting air and the other conducting blood must develop in a coordinated way to generate the millions of functional alveolar gas-exchange units [1], [2], [3].

Lung development in the mouse begins on embryonic day 9.5 (E9.5) when the lung primordium appears as a ventral bud in the primitive foregut [4]. Airway branching begins around E9.5–12 and continues through the “pseudoglandular” [E12–E16.5] and “canalicular” [E16.5–E17.5] stages. Thereafter, during the “saccular” stage [E17.5 to postnatal day 4 (P4)] the distal airways form the saccular units which are further subdivided by secondary *septae* formed during the alveolar stage (P4–P28 in mice) to form mature alveoli. This sequence of events is tightly controlled by the concerted action of growth factors, transcription factors, and mechanical forces [5], [6], [7].

Prominent role in the regulation of lung development and homeostasis is played by members of the Bone Morphogenetic Protein (BMP) family [8]. BMPs, like all other members of the TGFβ superfamily, signal via specific membrane receptors that have serine-threonine kinase catalytic activity [9]. Functional BMP receptor units are composed of two Type-I and two Type-II receptor polypeptides. Four different Type-I BMP receptors (ALK2, ALK3/BMPRIa, ALK6/BMPRIb and ALK1), and three Type-II receptors (BMPRII, ActRIIA and ActRIIB) have been identified [10]. Upon ligand binding, the constitutively active Type-II receptors phosphorylate and thus activate their Type-I partners, which in turn phosphorylate their intracellular targets, the receptor-regulated Smad proteins 1, 5 and 8. Phosphorylated Smads form complexes with the “common” Smad4 and translocate to the nucleus where they regulate expression of their target genes, synergistically with other transcription factors [8], [11]. BMPs can also signal via Smad-independent intracellular pathways that involve mitogen-activated protein (MAP) kinases [12], [13].

Several studies using transgenic and conventional or conditional knock-out mice have clearly demonstrated the key role played by BMPs during early lung development [14], [15], [16], [17], [18], [19], [20], [21]. Disruption of BMP signaling by ectopically expressing the BMP antagonists noggin or gremlin in the lung epithelium [15], [22], inactivating BMP receptors [16] or expressing a dominant negative form of the BMP Type-I receptor (dnALK6) result in abnormal distal lung architecture. Remarkably, over-activation of the BMP pathway is also incompatible with normal lung development. Ectopic over-expression of Bmp4 in the epithelium leads to smaller lungs and to substantially reduced epithelial cell proliferation [14] and mice with deletion of the BMP antagonist Follistatin-Like 1 (Fstl1) gene die at birth from respiratory distress and show multiple defects in lung development [23], [24].

Moreover, increasing evidence supports a key role for BMP signaling angiogenesis and vasculogenesis in the lung [25], [26]. A large number of genetically modified mice with lesions in genes encoding either BMP ligands, receptors or antagonists exhibit defective angiogenesis. The importance of BMP signaling for the vascular system is demonstrated by the association of mutations in genes encoding for BMPRII and ALK1 with the development of two genetic vascular diseases, namely, pulmonary arterial hypertension and hereditary hemorrhagic telangiectasia.

The BMP signaling pathway has also been implicated in the regulation of adult lung homeostasis and tissue repair following injury [27], [28], [29], [30], [31]. Several BMP ligands have been found up-regulated in allergen challenged lungs [27] and notably, ectopic expression of gremlin by adenovirus mediated gene transfer in the lung of adult rats causes severe pulmonary fibrosis [32] illustrating the importance of the BMP pathway for lung homeostasis as well. Consistently, over-expression of gremlin has been observed in humans suffering from Idiopathic Pulmonary Fibrosis (IPF) [28], [29].

Despite the significant progress, the precise mechanism(s) by which BMPs regulate lung development have not been fully delineated [5]. Moreover, characterization of the mechanisms by which BMPs affect adult lung homeostasis and tissue repair is still rudimentary. The current study was based on the premise that identification of the actual cellular targets of BMP mediated signaling during lung development, homeostasis and adult lung tissue repair will facilitate interpretation of earlier genetic studies, guide rational targeting of BMP signaling components in future experiments and contribute to the clarification of the mechanisms of action of this signaling system. Therefore, utilizing a mouse transgenic line carrying the eGFP reporter gene under the control of a canonical BMP pathway responsive regulatory element [33] we have constructed a detailed spatiotemporal map of canonical BMP signaling in the lung and identified putative cellular targets of this signaling pathway during lung development and adult lung tissue-injury repair. Our studies suggest that canonical BMP pathway plays key roles during early development of the pulmonary vasculature and the management of lung epithelial cell progenitors during late lung development and repair of adult lung tissue injury.

## Materials and Methods

### Ethics Statement

Animals were housed in individually ventilated cages under specific pathogen free conditions, in full compliance with FELASA (Federation of Laboratory Animal Science Associations) recommendations, at the Animal House Facility of the Foundation for Biomedical Research of the Academy of Athens (Athens, Greece). All procedures for care and treatment of animals were approved by the Institutional Committee on Ethics of Animal Experiments and the Greek Ministry of Agriculture (Permit Number: K/1054).

### Animals

BMP-responsive eGFP expressing (BRE-eGFP) mice [33] kept in the C57BL/6 background maintained on a 12∶12 hours light∶dark cycle at the Animal Facility of the Foundation for Biomedical Research of the Academy of Athens.

### Histology

Collection of fetal lung tissues was carried out at the embryonic (E11 and E12) pseudoglandular (E13.5 and E14.5), canalicular (E17) and saccular (E19.5) stages. Noon of the day of the vaginal plug was considered embryonic day (E) 0.5. Additionally, tissues were collected on post-natal days 1 (P1), 15 (P15) and 2 months after birth (adult). All embryonic and P1 lungs were removed surgically, cleaned from the surrounding tissues and placed in 4% paraformaldehyde (Merck, 104004) in PBS for 24 hours at 4°C. For analysis of P15 and adult BRE mice the right lung-lobes were used for RNA/Protein isolation, whereas the left lungs were gently perfused with a mixture of 4% PFA∶OCT (2∶1) using a 20G catheter (Abbocath G717-A01, 4535-20), the bronchus was ligated and placed in 4% PFA for 24 hours at 4°C. Thereafter, the tissues were placed in PBS with 30% sucrose for 24 hours at 4°C, washed with PBS, embedded in OCT (Shandon Cryomatrix) and kept at −80°C until use. Cryostat sections 6–10 µm (Leica CM3050S) were placed on polylysine or SuperFrost Plus slides (Menzel Glaser), kept at room temperature for 3 hours with silica gel (Merck, 101969) and then stored at −80°C. The primary antibodies used for immunostaining were: goat anti-CC10 (Santa Cruz, SC9773), rat anti-PECAM1 (CD31) (BD-Biosciences, 550274), rat anti-VEGFR2 (eBiosiences 14–5821), rabbit anti-SpC (Chemicon, AB3786), rabbit anti-CGRP (Sigma-Aldrich, C8198), mouse anti-α smooth muscle actin-Cy3 (Sigma-Aldrich, C6198), Syrian hamster anti-T1α (DSHB 8.1.1), Sheep anti-BrdU (Fitzgerald, 20-BS17), mouse anti-Foxj1 (generous gift of Dr. S. Brody), mouse biotinylated anti-Muc5ac (Abcam, ab79082), anti-SM22alpha (Abcam, ab14106) and chicken anti-GFP (Abcam, ab13970). The secondary antibodies used were: donkey anti-goat Texas Red (Jackson Immuno-research, 705-076-147), donkey anti-chicken FITC (Jackson Immuno-research, 703-096-155), goat anti-rat AlexaFluor594 (Molecular Probes, A11007), goat anti-rat AlexaFluor647 (Molecular Probes, A21247), goat anti-Syrian hamster AlexaFluor568 (Molecular Probes, A21112), goat anti-Syrian hamster AlexaFluor647 (Molecular Probes, A21541), donkey anti-rabbit Texas-Red (Jackson Immuno-research, 711-076-152), donkey anti-sheep AlexaFluor568 (Molecular Probes, A21099) and donkey anti-rabbit Cy5 (Jackson Immuno-research, 711-176-152). The slides were mounted using the ProLong Gold Antifade Reagent with DAPI (Molecular Probes, P36931). Images were captured with a Leica DMRA2 fluorescent microscope equipped with a Leica DFC320 and DFC350 FX digital cameras and a Leica TCS-SP5 confocal microscope (Leica Microsystems, Wetzlar, Germany). Image analysis was performed using Adobe Photoshop CS3, ImageJ 1.41 and Volocity LE.

### pSmad1/5/8 immuno-staining

Tissue sections prepared as described in the previous section were treated 1.5% H_2_O_2_ in MetOH for 30 min at RT in the dark to inactivate endogenous peroxidise. After blocking with 2% normal donkey serum (Sigma D9663) in TBS-Tween 0.3% for 2 hours at room temperature, the sections were incubated with rabbit anti-pSmad1/5/8 (Chemicon, ab3848) antibody overnight at 4°C in TBS-Tween 0.3% and then incubated for 1 hour at room temperature with the secondary anti-Rabbit HRP (Santa Cruz, SC2301). The slides were developed using DAB chromogen (DAKO K3468) development according to the manufacturer's instructions. Normal rabbit IgG fraction was used as control primary antibody (isotype control). Nuclei were counterstained with Mayer's Hematoxylene.

### RNA isolation and real-time quantitative PCR analysis

Total RNA from mouse lungs was isolated using the Tri-Reagent protocol (Sigma, St. Louis, MO) and the yield and purity of RNA was determined electrophoretically and spectrophotometrically. After DNase treatment with RQ1 RNase-Free DNase (Promega, M160A), 2 µg of RNA were reverse transcribed into cDNA using M-MLV Reverse Transcriptase (Promega, M170B) and random primers (Invitrogen, 58875) according to the manufacturer's instructions. The primer pairs for real-time PCR were designed using Beacon Designer v7.01 software (Premier Biosoft International, Palo Alto, CA). Sequences for the primer pairs used are given in table S1. The PCR reactions contained SYBR® Green ER™ qPCR Super Mix Universal (Invitrogen, 11762-500), 200 nM of each primer (Invitrogen, Carlsbad), and 0.2 µl of cDNA template in a 20 µl reaction volume. RT-qPCR cycling parameters were initial incubation at 50°C for 2 minutes, denaturation at 95°C for 10 minutes followed by 50 cycles of 15 seconds at 95°C and 40 seconds at 60°C. Data collection was carried out using a Chromo4 Real-Time PCR detector (BioRad) and analyzed with Opticon Monitor 3 expression analysis software (BioRad). Relative levels of mRNA expression were calculated according to the ΔΔCt method [34].

### Western Blot Analysis

For protein extraction cells were homogenized in lysis buffer containing 50 mM Tris-Cl pH7.6, 150 mM NaCl, 1% (v/v) Triton ×100, 5 mM EDTA pH 8.0, 1 mM PMSF, 1∶25 (v/v), Protease Inhibitor (Roche Applied Science, 11836153001), 2 mM Na_3_VO_4_, 5 mM NaF, 2 mM Sodium Pyrophosphate, 13.3 mM β-Glycerophosphate disodium salt. Homogenized cells were centrifuged at 13.000 rpm for 15 min at 4°C and protein samples were stored at −20°C until use. For detection of pSmad1/5/8 separated proteins were transferred to Immobilon-P membrane (Millipore, IPVH00010) and analyzed using rabbit anti-pSmad1/5/8 (Chemicon, ab3848, 1∶1000) coupled with the anti-rabbit HRP (Santa Cruz SC2301, 1∶5.000) and anti-β actin (Sigma-Aldrich, A5316, 1∶5.000) combined with the anti-mouse HRP (Sigma-Aldrich, A4416, 1∶40.000). Densitometry was done using the ImageJ 1.41 software.

### In-vivo lung tissue-injury/repair models

Female BRE-eGFP animals, between 12–16 weeks of age were treated with naphthalene or bleomycin. For naphthalene treatment, 300 mg/kg naphthalene in corn oil (vehicle) or vehicle alone was administered by intra-peritoneal injection. Lung tissues were collected on days 2, 3, 6, 9 post naphthalene-treatment. For bleomycin treatment, bleomycin (0.033 Units/mouse, Nippon Kayaku co ltd, Bleocin) diluted in PBS or PBS alone (vehicle) was administered intra-tracheally in 30 µl total volume. Lungs were collected on days 2, 4 and 12 post-bleomycin-treatment.

### Ex-vivo culture of fetal lung explants

Transgenic E12.5 lung explants were isolated and cultured, for 8 or 24 hours, in a 1∶1 mixture of DMEM: F12 (GiBCO, 11039), supplemented with Insulin-Transferrin-Selenium (Gibco, 51300-044) and antibiotics. Explants were placed on Nuclepore Track-Etched Membranes (Whatman, 110414) and they were incubated in 4-well plates (Thermo Scientific, 144444). LDN193189 (Axon MedChem, Axon 1509) and SB431542 (Sigma-Aldrich, S4317) were used at a concentration of 10 µM. Untreated and vehicle (DMSO, Sigma-Aldrich, D2650) treated lungs were used as controls. In some experiments, E12.5 lung explants were treated with 100 ng/ml BMP4 (Peprotech, 120-05ET), or 250 ng/ml FGF10 (Peprotech, 100-26) or 500 nM Sonic Hedgehog (Shh) agonist-Purmorphamin (Calbiochem, 540220) for 8 hours.

Endodermal buds and distal lung mesenchyme were isolated from E12.5 lungs as previously described [35], [36]. Briefly, isolated distal lung tips were treated for approximately 5 min with Trypsin 0.5% Pancreatin 2.5% in HBSS 1× (Gibco, 14170) on ice. Mesenchyme was removed using 26G needles and the epithelial buds as well as the isolated mesenchyme were placed in a (1∶1) mixture of Growth Factor Reduced Matrigel (BD Biosciences, 356230) and DMEM: F12 (GiBCO, 11039) 10% FCS (Gibco, 10500), Pen Strep (Gibco, 15070, 1∶100) and incubated for 8 hours at 37°C, 5% CO_2_.

### In vitro culture of primary adult lung cells

Adult primary lung cells were isolated as described in [37] with small modifications. Briefly, lungs were isolated, cut in small pieces and digested over-night at 4°C with 0.1% protease type-XIV (Sigma-Aldrich, P5149) in Joklik's MEM (Sigma Aldrich, 8028) containing antibiotics (Gibco, 15070). Lung pieces were transferred in Joklik's MEM supplemented with 10% FBS (Gibco 10500), pipetted gently several times to release cells and passed through 70 µm cell strainer (BD Biosciences, 352350). Cells were washed with MCDB-201 (Sigma-Aldrich, M6770) containing Insulin-Transferrin-Selenium (Gibco, 51300-044) and viability was measured using Trypan Blue. 10^6^ cells (10^6^/ml) were cultured in 12-well plates (Nunc, 150628) coated with Bovine collagen (Nutacon, 5409) for 24 hours. Coating was done with 100 µg/cm^2^ collagen, for 1 hour at 37°C. Thereafter, cultures were rinsed with MCDB-201 to remove detached cells and 500 µl MCDB-201 with Insulin-Transferrin-Selenium and 1 ng/ml EGF (Peprotech, 315-09) was added. After 1 week, colonies of epithelial cells were formed and infected with recombinant adenoviruses expressing either constitutively-activated ALK3 (caALK3), caALK5, Smad1 or Smad3. After 48 hours incubation the treated cells were used either for cytochemistry or for RNA extraction.

### Isolation of lung subpopulations by FACS

P3 mice were anesthetized, the abdominal cavity was opened and ventricular vein and artery were cut. The chest was opened and the right atrium nicked. Lungs were perfused through the right ventricle of the heart with 1 ml ice-cold 1× PBS using a 26G syringe, cut in small pieces using a razor blade and digested with the Enzyme mix [Elastase (Sigma-Aldrich, 45124): 3 U/ml, Collagenase: 0.1 U/ml, Dispase: 0.8 U/ml (Roche Applied Science, 11097113001) and DnaseI 50 µg/ml (Sigma-Aldrich, DN25] in PBS, at 37°C for 1 hour with rotation. Equal volume of HBSS^++^ [HBSS 1× (Gibco, 14170), supplemented with 2% FCS (Gibco, 10500), 0.1 M HEPES (Sigma-Aldrich, H0887) and 15% Cell Dissociation Buffer (Gibco, 1315-016) or EGTA 2 mM] was added. Cells were gently agitated several times, passed through a 40 µm cell strainer (BD Biosciences, 352340) and centrifuged 10 minutes at 1200 rpm, at 4°C. The supernatant was discarded and cells were re-suspended in HBSS^++^ and counted with a hemocytometer. Specific subpopulations of lung cells were isolated using a BD FACS-Aria IIu cell sorter by staining the isolated lung cells as previously described by Teisanu et al. [38] with biotinylated anti-CD31 (BD Biosciences, 553371), anti-CD45 (Biolegend, 103103) and anti-CD34 (eBioscience, 13-0341-85) combined with Streptavidin APCCy7 (Biolegend, 405208), with anti-Sca1-Alexa Fluor647 (Biolegend, 108118) andanti-EpCamPECy7 (Biolegend, 118216). DAPI was included for dead cell exclusion. The EpCam^pos^-CD31^neg^-CD45^neg^-CD34^neg^ cells were separated into Sca1^neg^ and Sca1^low^ and each of these two populations were separated by sorting into eGFP^neg^, eGFP^low^ and eGFP^high^. Isolated cells were used either for further *in-vitro* cultivation, RNA expression or Western Blot analysis.

In vitro cultivation of sorted epithelial cells in Matrigel was done as previously described [38] with minor modifications. 50 µl of 2×10^3^ sorted cells and 2×10^5^ mouse lung stroma cells (MLg) were mixed with equal volume of Growth Factor Reduced Matrigel (BD Biosciences, 356230). Cells suspended in Matrigel were added to the chamber of 24-well Transwell filter inserts (BD Biosciences, 353095) and placed in 24-well, flat-bottom, culture plates containing DMEM: F12 culture medium (GiBCO, 11039) supplemented with 5% FCS (Gibco, 10500), Insulin-Transferrin-Selenium (Gibco, 51300, 1∶100), Pen Strep (Gibco, 15070, 1∶100), amphotericin B (Gibco, 15290, 1∶1000) and 10 µM SB431542 (Sigma-Aldrich, S4317). Culture medium was changed every other day until day 8. SB431542 was then removed and the cultures were maintained for an additional four days before fixing overnight at 4°C with 4% PFA in PBS. The Matrigel was removed by freezing-thawing and rinsing with fresh PBS. The content of the Transwell inserts was embedded in OCT (Shandon Cryomatrix) and 10 µm sections were prepared by cryostat (Leica CM1950).

Although epithelial colonies developed in Matrigel even in the absence of SB431542 its presence resulted in higher number of larger colonies and therefore this culture system was preferred. Treatment of the Mlg cells with SB431542 for 24 hours led to substantial increase in FGF10 mRNA synthesis (data not shown) suggesting that stroma cell derived FGF10 may be part of the underlying mechanism.

### Statistical analysis

One way analysis of variance (One-way ANOVA) combined with “Bonferoni's Multiple Comparison Test”, was done using GraphPad Prism. (*/^#^) P<0.05, (**/^##^) P<0.01, (***/^###^) P<0.001.

## Results and Discussion

### Spatiotemporal pattern of expression of the BRE-eGFP reporter during lung development

**Figure 1:**
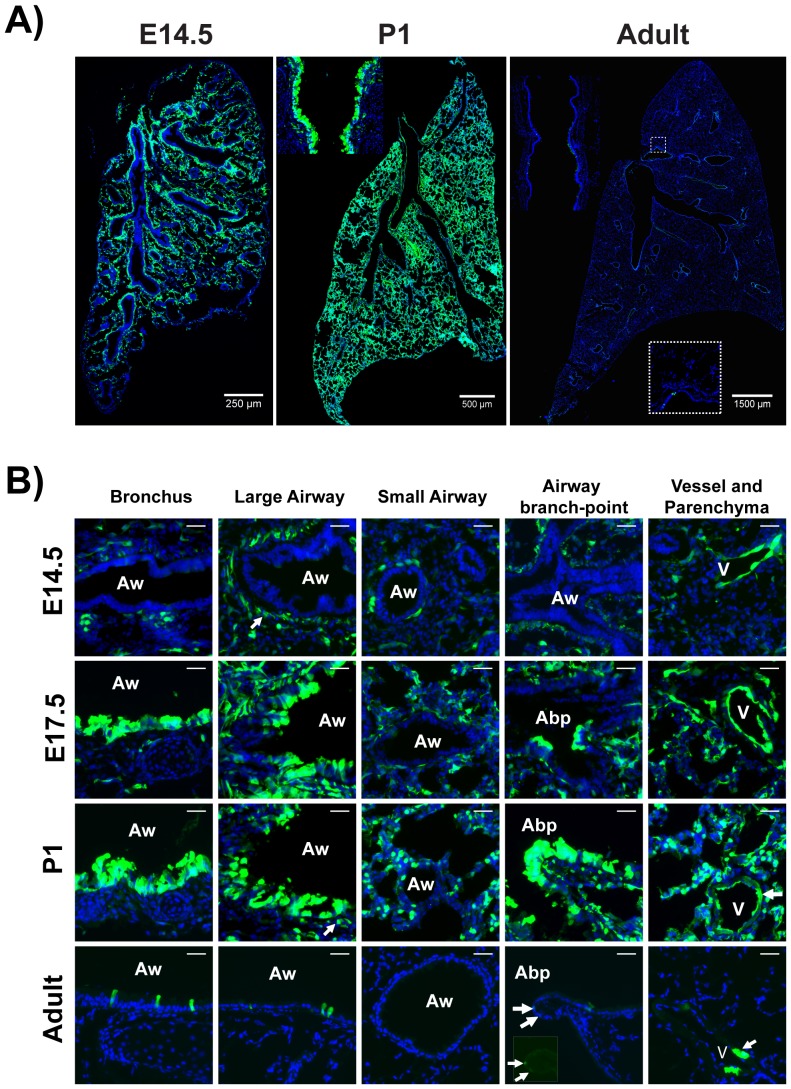
Pattern of eGFP expression in BRE-eGFP transgenic mouse lungs. A) Panoramic pictures produced by digitally merging several images from E14.5 (merge of two images taken at 10×), P1 (merge of fifty-seven images taken at 20×) and adult lung (sixty-four images taken at 10×). Insert in the P1 lung illustrates the high eGFP expression in trachea. Insert in adult lung image illustrates the small number of eGFP^pos^ cells in a large airway. B) High magnification images of E14.5, E17, P1 and adult lung tissue-sections. “Aw” stands for airways, “V” for vessels, and “Abp” for airway branch-points. Insert in adult airway branch-point image depicts the small number of eGFP^pos^ cells found in these locations. Nuclei are stained with DAPI (blue-staining).Scale bars: 30 µm.

Previous studies utilizing two independent canonical BMP pathway reporter mouse lines, one harboring a BRE-eGFP [33] and another harboring a BRE-lacZ allele [39] have demonstrated expression of the BRE-eGFP and BRE-lacZ reporters respectively in the epithelium of trachea and primary-bronchi already at around E10–E12.5. To extend these findings and map in detail the spatiotemporal pattern of canonical BMP signaling in the lungs, we collected lung tissues from the BRE-eGFP reporter mice at embryonic days E11, E12, E13.5, E14.5, E16, E17.5, E19.5 and postnatal days 1, 15 and 2 months and analyzed tissue sections for eGFP expression. Our analysis demonstrated that the BRE-eGFP reporter was active already at E11, reached maximal levels around birth, thereafter declining to minimal levels in the adult (Figure 1 and Figure S1).

Around E11, eGFP expression was confined to the endothelial cells of the large pulmonary vessels, and the developing vascular network in the parenchyma. A very small fraction of sub-epithelial smooth muscle cells (SMCs) expressed very low levels of eGFP at E12 (Figure S1). However, from E13.5 onwards robust eGFP expression was detected in sub-epithelial SMCs located at the proximal regions of the developing airways (Figures 1 and Figure S1 and S2).

From E16 onwards, expression of eGFP was detected in both proximal and distal epithelial compartments.In the proximal epithelium (i.e. the developing airways) eGFP expression exhibited a proximal-distal axis pattern, with high eGFP expression in bronchi and large airways and surprisingly no expression at the distal portion of the growing airway tree. Interestingly, isolated clusters of eGFP^pos^ cells were observed at the tips of airway branch-points. In the distal lung compartment, eGFP expression was detected in cuboidal epithelial cells of the developing saccules (Figure 1B). As lung morphogenesis approached completion, the eGFP signal was reduced and in adult mice,(∼2 months old), few eGFP^pos^ cells were detected scattered in the epithelium of bronchi and large airways, in the alveolar compartment and in a subpopulation of cardiac cells in the outer layer of the *tunica media* of the pulmonary veins (Figure 1B) [40].

### Expression of the BRE-eGFP reporter in the developing lung mirrors faithfully activation of the canonical BMP pathway

**Figure 2:**
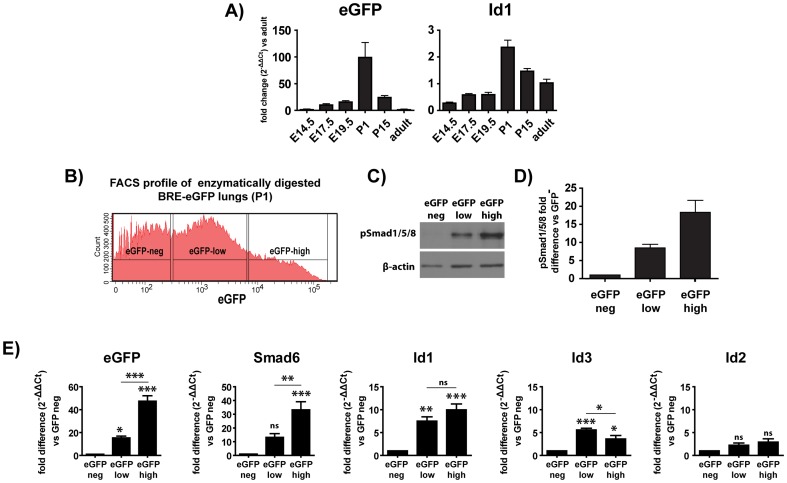
Expression of the BRE-eGFP reporter in the developing lung mirrors faithfully activation of the canonical BMP pathway. A) Kinetics of eGFP and Id1 mRNA expression levels during lung development analysed by Real-Time PCR. Values represent mean ± SEM of five individually analyzed animals. B) eGFP profile of enzymatically digested P1 lungs. The gates used to separate lung cells by sorting into eGFP^neg^, eGFP^med^ and eGFP^high^ are indicated. C) Representative Western-blotting of proteins prepared from sorted populations with an anti-pSmad1/5/8 antibody. D) Quantitation of the pSmad1/5/8 relative to the eGFP^neg^ group.Values represent mean ± SEM of two independent experiments. E) Analysis of mRNA expression levels for eGFP, Smad6, Id1, Id3 and Id2in sorted cells. Values represent mean ± SEM of seven individually analyzed experiments. Groups were compared using one-way analysis of variance (ANOVA) with Bonferonis's post-hoc analysis. *P<0.05, **P<0.01 and ***P<0.001.

**Figure 3:**
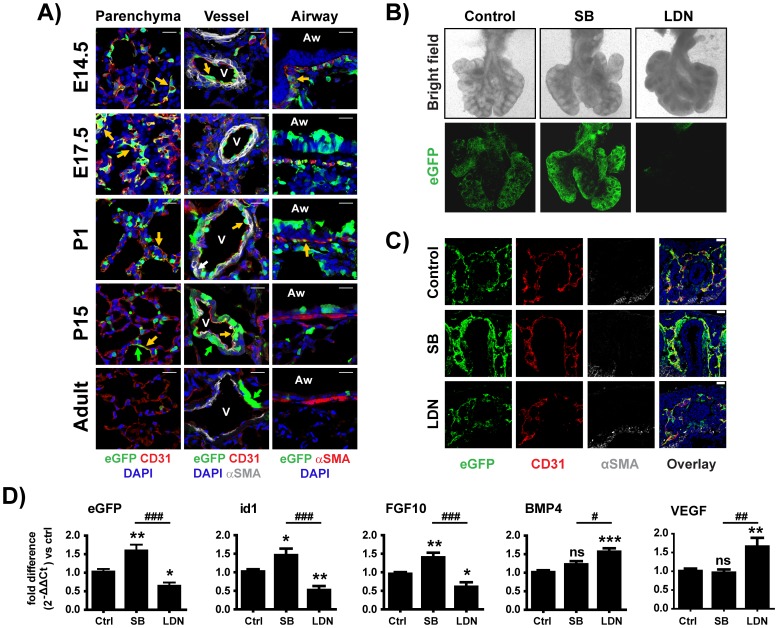
Canonical BMP-pathway is active in the developing vascular and sub-epithelial smooth muscle networks. A) Confocal images of tissues from different developmental stages stained with antibodies to eGFP and CD31 (parenchyma and blood-vessels), and eGFP and αSMA (blood-vessels and large-airways). Nuclei were counterstained with DAPI. Representative eGFP^pos^ cells in the different tissue compartments are shown with arrows. Green arrows depict eGFP^pos^ cells, whereas yellow arrows depict eGFP^pos^-CD31^pos^ or eGFP^pos^-αSMA^pos^ cells. A white arrow in the P1 vessel indicates a rare eGFP^pos^-αSMA^pos^ vascular smooth-muscle cells. “Aw” defines airways and “V” vessels. Scale bar: 20 µm. B) Whole E12 lung-explants, cultured on Nuclepore membranes, were treated with vehicle, SB431542, or LDN193189. Upper panel shows transmitted light images of representative explants. Lower panel shows eGFP expression in the same explants. C) Confocal images of tissue-sections from lung-explants treated with vehicle, SB431542 or LDN193189 for 8-hours and stained with antibodies to eGFP, CD31 and αSMA. Nuclei were counter-stained with DAPI. D) Quantitative-PCR analysis of eGFP, Id1, FGF10, BMP4 and VEGFα mRNA expression in vehicle or inhibitor-treated lung explants. Values represent mean ± SEM of six SB431542-treated, seven LDN193189-treated and thirteen vehicle treated, individually analyzed explants per group, compared using one-way analysis of variance (ANOVA) with Bonferoni's post-hoc analysis. *P<0.05, **P<0.01 (vehicle *vs* inhibitor treated groups) and ^#^P<0.05, ^##^P<0.01, ^###^P<0.001 (SB *vs* LDN treated groups).

Although the capacity of BRE element to monitor BMP mediated transcriptional activation *in-vivo* has been demonstrated previously [33], [39], [41], additional studies were undertaken to further increase confidence with respect to transgene expression in the lung. The kinetics of mRNA expression for eGFP and the BMP target gene Id1 were analyzed by quantitative PCR. EGFP and Id1 mRNA levels followed similar kinetics to eGFP protein expression, reaching maximum levels around birth (Figure 2A). P1 lungs were therefore enzymatically digested and lung cells were separated by cell sorting on the basis of eGFP expression into eGFP negative (eGFP^neg^), eGFPlow (eGFP^low^) and eGFP high (eGFP^high^) cells (Figure 2B). Western blot analysis of protein extracts from the isolated population demonstrated good correlation between expression of eGFP and levels of pSmad1/5/8 (Figure 2C, D). Analysis of the mRNA levels for eGFP and BMP-regulated target genes such as Smad6, Id1 and Id3 demonstrated very good correlation between the level of eGFP expression and the activation of known BMP targets (Figure 2E). Interestingly, expression of Id2 mRNA did not correlate with levels of eGFP expression. Furthermore, treatment of embryonic lung explants *ex-vivo* with either BMP- or TGFβ-receptor inhibitors resulted in either suppression or augmentation of BRE-eGFP transgene expression, respectively (see below Figure 3B). Moreover, treatment of embryonic lung explants with FGF10, a known inducer of BMP4 production by distal endodermal cells, or recombinant BMP4 resulted in substantial up-regulation of eGFP expression (Figure S3). Treatment of the lung explants with purmorphamin, a Shh agonist, resulted in redistribution of the eGFP staining pattern that involved a small increase of eGFP expression in a narrow zone of sub-mesothelial cells and a reduction of eGFP expression in the more central parts of the mesenchyme. This pattern of expression could stem from a Shh mediated reduction in FGF10 synthesis by mesenchymal cells [35] that in turn leads to a more confined BMP expression zone.

Too further validate the BRE-eGFP reporter animals and assess its sensitivity in comparison to staining tissues for nuclear pSmad1/5/8 by immuno-histochemistry, we stained adjacent tissue sections of P1 lungs, the developmental stage demonstrating maximal BRE-eGFP reporter activity, with anti-eGFP and anti-pSmad1/5/8 antibodies. As shown in Figure S4, nuclear pSmad1/5/8 was detected in regions with intense eGFP immuno-staining demonstrating consistency between BRE-eGFP reporter activity and pSmad1/5/8 immuno-staining (figure S4). Unfortunately, in our hands the anti-pSmad1,5,8 reagents we have analyzed so far were unable to detect pSmad1/5/8 in lung tissues from embryos before stage E19.5 and thus we were not able to confirm correlation of eGFP and pSmad1/5/8 at earlier developmental stages. A number of studies have indicated that the value of measuring pSmad1/5/8 as the sole indicator for canonical BMP pathway activation is questionable since presence of pSmad1/5/8 does not necessarily lead to transcriptional activity of BMP target genes. Canonical BMP pathway activity is known to be regulated by cell type-specific Smad-interacting transcription factors, co-activators and co-repressors. For example Tbx1 can bind to Smad1, interfere with Smad1/Smad4 interaction and decrease BMP transcriptional activity without affecting the amount of pSmad1/5/8 in the responding cell [42]. Furthermore, Leeuwis et al. [41] demonstrated that in the kidney of BRE-eGFP animals, whereas the expression pattern of BRE-eGFP exhibited excellent correlation with the expression of the BMP7 and the BMP target genes Id1, and Smad6, in contrast pSmad1/5/8 exhibited a brought expression pattern that did not correlate with the expression of BRE-eGFP, BMP7, Id1 or Smad6. Therefore, a safer assessment of canonical BMP pathway should combine analysis of several known BMP target genes and pSmad1/5/8 immuno-stainings when this is technically possible. Collectively, the analysis by Monteiro et al. [33], Leeuwis et al. [41] and the results described herein indicate that the BRE-eGFP transgenic line reports quite faithfully activation of the canonical BMP pathway *in-vivo* and *ex-vivo* and thus it provides an additional method for assessing potential canonical BMP pathway activation.

### Canonical BMP-pathway is activated in the developing vascular and sub-epithelial smooth muscle networks during early lung development

To identify the putative cellular targets of canonical BMP signaling, tissue sections collected from lungs at different developmental stages were stained with antibodies specific for appropriate cellular markers. Double immunostaining for eGFP and CD31 (PECAM-1), a surface marker of mature endothelium, or VEGFR2, a surface marker of vascular progenitor cells (Figure S1), demonstrated that the majority of CD31^pos^ or VEGFR2^pos^ cells at E12, E14.5 and E17 were also eGFP^pos^ (Figure 3A), confirming that the network of eGFP^pos^ cells in the parenchyma is composed of vascular cells and that the canonical BMP pathway is activated during the establishment of the vascular network in the developing lung. At P1, ∼40% of the CD31^pos^ cells were eGFP^pos^, from P15 onwards the number of eGFP^pos^-CD31^pos^ cells declined and in the adult they were undetectable. EGFP expression with similar kinetics was observed in the endothelial cells lining the large blood vessels (Figure 3A). Very few eGFP^pos^ alpha smooth muscle actin (αSMA) double positive cells were found in the walls of the vessels (Figure 3A, white arrow in P1 vessel).

To validate the functional significance of BMP signaling for the development of the vascular network of the developing lung, *ex-vivo* cultures of E12 lung explants were treated with the respective BMP- or TGFβ/Activin-receptor inhibitors LDN193189 and SB431542. Treatment of lung explants for 24 hours with SB431542 led to a substantial increase in eGFP, Id1, and FGF10 mRNA expression, whereas treatment with LDN193189 led to a decrease ineGFP, Id1 and FGF10 mRNA expression and an increase, probably compensatory, in BMP4 and VEGFα mRNA expression (Figure 3B and D). Remarkably, even 8 hours of treatment with the SB431542 led to an increase in the density of the CD31^pos^ network and a more pronounced increase in the eGFP^pos^ network, whereas, similar treatment with LDN193189 led to reduction in the density and connectivity of the eGFP^pos^ and CD31^pos^ cells (Figure 3C).

Collectively, these findings indicate that during the pseudoglandular stage, in the course of which most of the stereotypical branching morphogenesis is thought to take place, an important target of canonical BMP signaling in the lung is the developing pulmonary vasculature.

Strong eGFP expression was detected in the developingsub-epithelial SMC layer of the proximal airways from E14.5 to P1 (Figure 3A and Figure S1 & S2). The eGFP expression, which interestingly involved only a portion of the sub-epithelial SMCs, was undetectable by P15. It has been proposed that sub-epithelial SMCs originate from a pool of sub-mesothelial, FGF10-expressing, mesenchymal cells. Under the influence of several signalling pathways, such as those mediated by FGF, BMP, Shh, and Wnt, these cells proliferate and passively translocate to surround the proximal portion of the developing airway tree [35], [43], [44], [45], [46]. http://www.sciencedirect.com/science/article/pii/S0012160611010189 - bb0125To map more precisely the temporal activation of the BRE-eGFP reporter during development of the sub-epithelial SMC compartment we isolated P11, P12 and P13.5 lungs from the BRE-eGFP reporter animals and analysed co-expression of eGFP and SM22a, a marker of both primitive and mature SMCs [47], or αSMA a marker of more differentiated SMCs. As shown in Figure S1B, SM22α expression was observed in a sub-epithelial population of cells around the developing proximal airways already at embryonic stage E11, αSMA expression was evident only in the most proximal portion of the SM22a^pos^ zone and eGFP expression was clearly detected in the αSMA^pos^ zone only after embryonic stage E13.5 (very few αSMA^pos^ cells expressed very low levels of eGFP at E12). These findings are consistent with the notion that canonical BMP signalling does not involve primitive SMCs but rather mature αSMA expressing sub-epithelial SMCs.

### Canonical BMP pathway activation in developing airway epithelial cells

**Figure 4:**
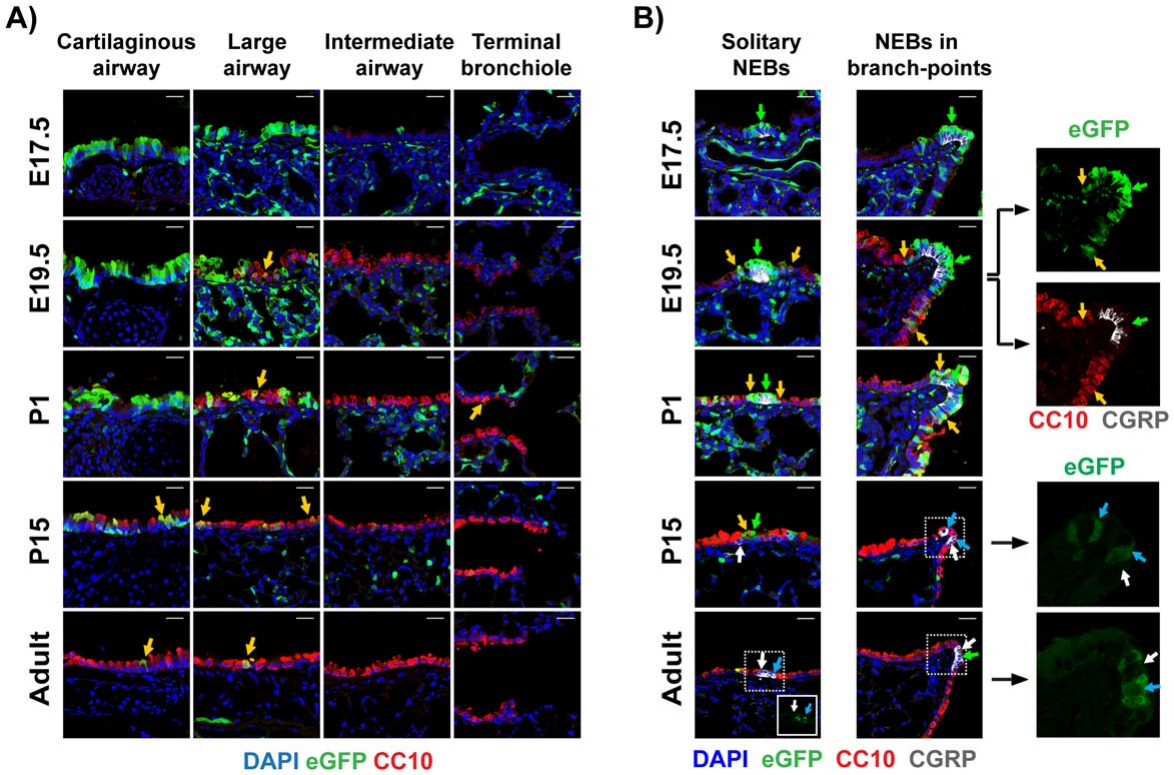
Activation of canonical BMP pathway in developing airway epithelial cells. A) Confocal images of tissue sections of lungs collected from E17, E19.5, P1, P15 and adult BRE-eGFP animals, stained for eGFP (green-staining) and CC10 (red-staining). Yellow arrows depict eGFP^pos^-CC10^pos^ epithelial cells found in the borders between the proximal eGFP^pos^ zone (cartilaginous and large-airways) and the distal CC10^pos^ zone of the airways (intermediate airways and terminal bronchioles). Nuclei were counter-stained with DAPI.Scale bar: 25 µm. B) Similar tissue section as above stained for eGFP, CC10, CGRP and DAPI demonstrating eGFP and CC10 staining around solitary and airway branch-point associated NEBs. Colors are as labeled in the figures. Green and yellow arrows depict representative eGFP^pos^-CC10^neg^ and eGFP^pos^-CC10^pos^ epithelial cells respectively. White and blue arrows in the P15 and adult lung sections depict representative eGFP^neg^-CGRP^pos^ and eGFP^pos^-CGRP^pos^ neuro-endocrine cells respectively. The eGFP and CC10/CGRP channels of the E19.5 airway branching point are shown separately to illustrate the CC10^low^ phenotype of the NEB's eGFP^pos^ epithelial “cap”. Cropped images of the eGFP channel illustrate the eGFP^pos^-CGRP^pos^ cells in the adult solitary NEB and in the P15 and adult branching point associated NEBs. Scale bars: 25 µm.

Although strong BRE activity was detected in the trachea and primary bronchi as early as E10–E12.5 [33], consistent with the demonstrated role of BMP signaling in trachea development [48], [49], BMP-driven eGFP-expression was not detected in the epithelial compartments of the interlobular airways before the canalicular stage of lung development. As shown in Figures 1 and 4A, minimal eGFP expression was detected around E14.5 in the large airways, and only after E17 strong eGFP signal was detected in cartilaginous and large airways following a proximal-distal axis pattern, with more intense signal in the proximal part of the bronchial tree. Immunostaining of BRE-eGFP lung tissue sections with an antibody specific for the Clara cell specific differentiation marker Scgb1a1/CCSP/CC10 (hereafter referred to as CC10), revealed expression of the eGFP reporter from E17.5 and onwards, in luminal cells of the developing airways. Strong CC10 expression was detected around E19.5, approximately two days after the onset of eGFP expression in the airway epithelium (Figure 4A) CC10 and eGFP exhibited partially overlapping zones of expression, with eGFP being highly expressed in the proximal portion and CC10 highly expressed in the distal portion of the developing airway tree. A zone of eGFP^pos^-CC10^pos^ cells appeared to separate the CC10^pos^ epithelial population from the eGFP^pos^ airway regions. Postnatal expansion of CC10^pos^ regions in the conducting airways was accompanied by a gradual reduction of the eGFP^pos^ regions (Figure 4A).

Interestingly, regions of airways exhibiting enhanced eGFP expression were detected close to the tips of airway branch-points in close proximity to neuro-epithelial bodies (NEBs). Analysis of the eGFP expression in relation to CC10 and CGRP, a differentiation marker of neuro-epithelial cells, revealed that eGFP^pos^-CC10^low^cells formed “caps” over clusters of CGRP^pos^ cells. From E19.5, the eGFP^pos^-CC10^low^“cap” cells (green-arrows in Figure 4B) were separated from the eGFP^neg^-CC10^pos^ cells by a zone of double positive epithelial cells (yellow-arrows in Figure 4B). Notably, the zone of eGFP^pos^-CC10^pos^cells coincided with zones richin NEBs (Figure S5). From P15 and beyond, the only NEB-associated eGFP^pos^ cells were CGRP^pos^ neuro-epithelial cells (blue-arrows in Figure 4B).

As the majority of eGFP^pos^ cells did not express the marker for secretory airway epithelium (CC10), their possible relationship to ciliated airway epithelial cells was investigated by staining with antibodies for Foxj1 a ciliated cell specific differentiation marker. As shown in Figure S6, a remarkably small portion of eGFP^pos^ cells were FoxJ1^pos^ (yellow-arrows in Figure S6).

Two alternative models have been proposed regarding the role of BMP signalling during lung branching morphogenesis. One model suggests that mesenchymal derived FGF10 induces Bmp4 expression by the distal bud epithelium to limit, in an autocrine manner, Fgf10-mediated bud outgrowth [45], [50]. The other model, based on the differential response of the distal epithelial buds to BMP4 in the presence or absence of mesenchyme, proposes that Bmp4 produced by the distal epithelium acts in an autocrine manner to limit proliferation of distal epithelial buds, and in a paracrine manner, on the adjacent mesenchyme, to induce production of a mesenchymal signal that enhances proliferation of distal epithelial buds [51]. Thus, according to the latter model the negative or positive effect of BMP signalling on branching is the outcome of a dynamic balance between negative autocrine and positive paracrine mechanism.

**Figure 5:**
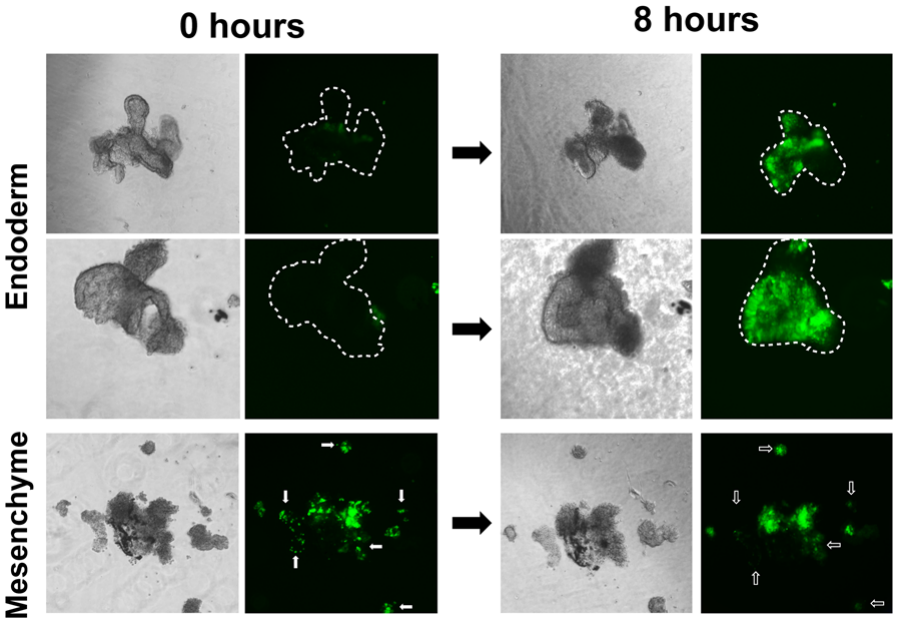
The BRE-eGFP reporter is activated in mesenchyme-free epithelial buds cultured in Matrigel. Distal endodermal buds and mesenchyme were isolated by micro-dissection from E12.5 BRE-eGFP embryos and cultured for 8 hours in Matrigel. Bright field and fluorescence images from the same colonies were acquired at time 0 and 8 hours. Note the strong re-activation of the BRE-eGFP reporter in mesenchyme-free endodermal buds and the reduced (diffuse) eGFP expression in the isolated mesenchyme after 8 hours in culture. Dotted lines indicate the location of the epithelial buds in the eGFP images and small clusters of cells with reduced eGFP expression are indicated with arrows.

The absence of eGFP reporter activation in the distal buds of the branching airway tree (Figures 1 and 4) prompted us to investigate whether this is the result of an intrinsic inability of the distal epithelium to activate the canonical BMP pathway or, in accordance to the second model, the result of a dynamic interplay with the adjacent mesenchyme. Therefore, mesenchyme free distal epithelial buds and epithelial free mesenchyme were isolated as previously described [35], [36] and cultured in Matrigel. Remarkably, within 8 hours of incubation, the BRE-eGFP reporter was strongly up-regulated in the mesenchyme-free epithelial buds and conversely it exhibited a tendency for decline in the isolated mesenchyme (Figure 5). These findings are consistent with the model of Bragg et al. [51] and moreover suggest that the elusive, mesenchyme derived, epithelial growth promoting signal could simply act by negatively regulating activation of the canonical BMP pathway in the distal epithelium buds.

Collectively, the above findings demonstrate that activation of the canonical BMP pathway in airway epithelial cells coincides with the beginning of the canalicular stage, when the developmental plan of the lung shifts from branching morphogenesis to the development of distinct respiratory epithelial cell compartments [52]. Keeping in mind that gas conducting airways and blood conducting vessels need to develop in a coordinated manner and be properly juxtaposed to support optimal gas-exchange in the adult, it is understandable that the tips of developing airways should reciprocally exchange growth, differentiation or guidance signals with the developing vasculature and other mesenchymal cells to ensure coordinated development and spatial integration of these two branching systems. The finding that disruption of the vascular network in growing lung explants affects branching morphogenesis by reducing primarily “orthogonal bifurcations” of the epithelial tubules [53], [54] and the findings presented herein demonstrating the influence of the adjacent mesenchyme on the activation of the canonical BMP pathway reporter (Figure 5) illustrates the importance of the crosstalk between epithelial and vascular cells during early lung development.

It should be pointed out that due to our failure to successfully apply anti-pSmad1/5/8 immuno-histochemistry in tissues from embryos earlier than stage E19.5, we cannot conclusively rule out for the moment activation of the canonical BMP pathway at the tips of the early branching endoderm. Further studies will be required to assess whether the BRE-eGFP reporter is not active in the distal endoderm because: a) non-canonical BMP pathways are primarily involved; b) the canonical BMP pathway is active however, does not reach the activation threshold required to activate the BRE-eGFP reporter; or c) that the dynamic balance between the mesenchyme and endoderm which keeps the BRE-eGFP reporter inactive most of the time (Figure 5), allows at critical points along the branching process transient spikes of canonical BMP pathway signaling to reach the nucleus and regulate expression of target genes.

### Canonical BMP pathway activation in alveolar epithelial cells

**Figure 6:**
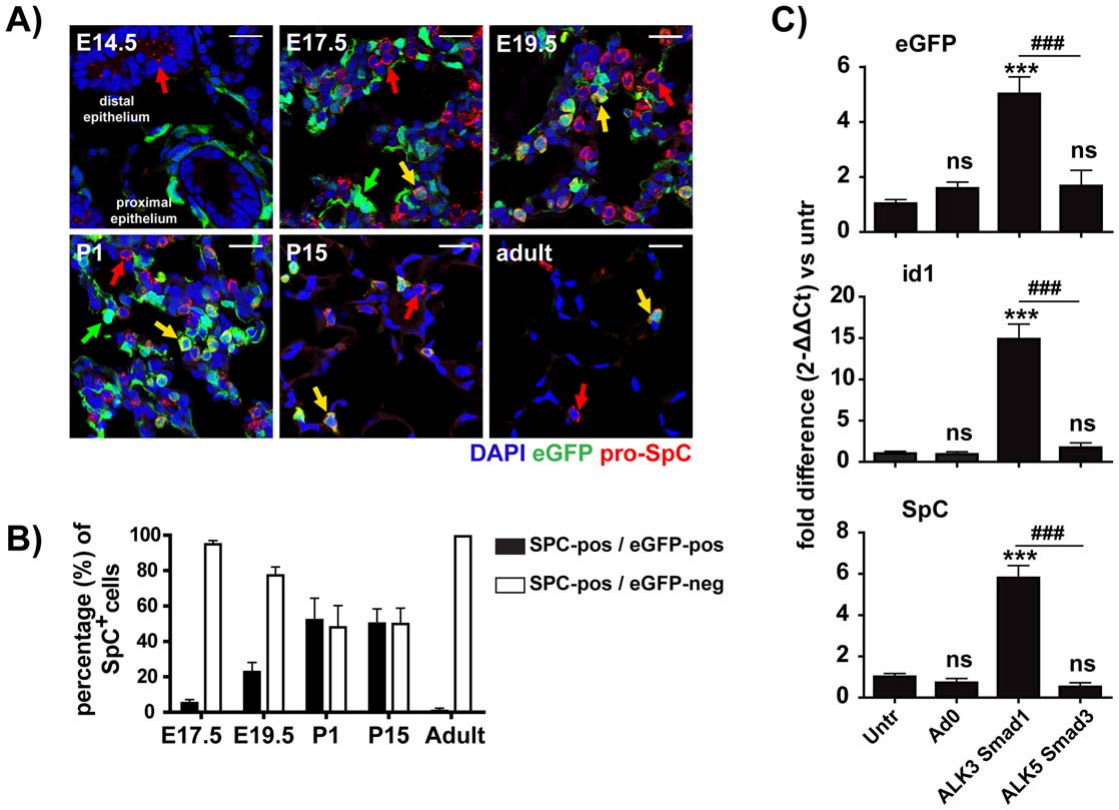
Canonical BMP pathway activation in alveolar epithelial cells. A) Confocal images of lung tissue-sections collected from E14.5, E17, E19.5, P1, P15 and adult BRE-eGFP transgenic animals stained for eGFP and pro-SpC. Red and yellow arrows depict eGFP^neg^-SpC^pos^ and eGFP^pos^-SpC^pos^ cells respectively. The sections were counterstained with DAPI. Scale bar: 20 µm. B) Morphometric analysis of SpC^pos^-eGFP^neg^ and SpC^pos^eGFP^pos^ cells in distal lung compartment. C) Adult lung cells were treated with combinations of ALK3 and Smad1, or ALK5 and Smad3 adenoviruses. Messenger RNA levels of eGFP, Id1 and SpC were analyzed 48 hours later by quantitative-PCR. Values represent mean ± SEM of 8 individually analyzed cultures per group compared to the untreated group using one-way analysis of variance (ANOVA) with Bonferonni's post-hoc analysis. ***P<0.001 and ^###^P<0.001 (ALK3/Smad1 *vs* ALK5/Smad3).

Over-expression of BMP ligands or inactivation of BMP receptors in the distal part of the developing lung leads to dramatic defects in alveolar development [14], [16], [18], [19]. Since eGFP expression was highly increased in lung parenchyma of the BRE-eGFP mice during the saccular and alveolar stages, the period during which functional alveoli are formed, we characterized the nature of the eGFP^pos^ cells in this part of the developing lung. Tissue sections from different developmental stages were analyzed by co-staining for eGFP and pro-surfactant protein C (pro-SpC), a marker of type-II pneumocytes. As shown in figure 6 and Figure S2, eGFP staining during the pseudoglandular stage was confined to endothelial cells and peri-bronchial smooth muscle cells of the proximal airways. Pro-SpC^pos^ epithelial cells in the distal airways were uniformly eGFP^neg^ at this developmental stage (Figure S2).In contrast, starting at the canalicular stage (E17) and persisting until approximately P15, a population of eGFP^pos^-pro-SpC^pos^ cells emerged in the distal lung compartments (Figure 6A).The SpC^pos^ cells at this stage of development are thought to be immature type-II pneumocytes [55]. Morphometric analysis demonstrated that during the saccular and alveolar stages, approximately half of the pro-SpC^pos^ alveolar cells were eGFP^pos^. Adult tissues, in which alveolarization was complete, contained a very small number of eGFP^pos^-pro-SpC^pos^ alveolar cells (∼0.5–1%) (Figure 6B). High resolution confocal microscopy analysis revealed that at all the developmental stages analyzed only a very small portion of type-I pneumocytes were eGFP^pos^ (Figure S7). Interestingly, ectopic over-expression of a constitutive ALK3 in cultures of adult primary pulmonary cells resulted in increased pro-SpC (∼6-fold) and Id1 (∼15 fold) mRNA levels, in comparison to the untreated or ALK5ca treated groups (Figure 6C) further implicating canonical BMP-signaling in the physiology of type-II pneumocytes.

The sequential appearance of eGFP^neg^-SpC^pos^ (immature type-II pneumocytes), eGFP^pos^-SpC^pos^ and eGFP^neg^-SpC^pos^ (mature type-II pneumocytes) may suggest that the eGFP^pos^-SpC^pos^ population is an intermediate step through which immature type-II pneumocytes must pass before developing into mature functionally competent type-II pneumocytes. This conclusion is compatible with the finding that mice with canonical BMP pathway disrupted due to deletion of the Smad1 gene accumulate in their developing lungs high numbers of periodic acid-Schiff (PAS) positive immature type-II pneumocytes [19].

The above findings indicate that during the cannalicular stage, an important target of canonical BMP signaling in the distal lung compartment is the developing type-II pneumocyte population. This could explain the dramatic distal lung phenotype of animals with disrupted BMP signaling in the distal lung epithelium [15], [16], [18], [19].

It is very intriguing that with the beginning of the canalicular stage, when the developmental plan of the lung shifts from branching morphogenesis to the development of distinct respiratory epithelial cell compartments [52], the BMP-responsive eGFP reporter is activated in two epithelial domains, namely, the proximal airways and the distal developing alveolar sacs. The conclusion that canonical BMP signaling becomes crucial for lung epithelial cell development at the cannalicular stage is consistent with earlier genetic studies demonstrating that deletion of Bmpr1 [16], [18] or Smad1 [19] and ectopic expression of negative regulators of BMP signaling such as XNoggin and dominant-negative ALK6 [15] by SpC-promoter-driven expression, resulted in defects in lung development visible only around E15-E16, i.e. after completion of the branching morphogenesis.

### The eGFP^pos^ epithelial compartment is enriched for epithelial progenitors capable in forming colonies in Matrigel

**Figure 7:**
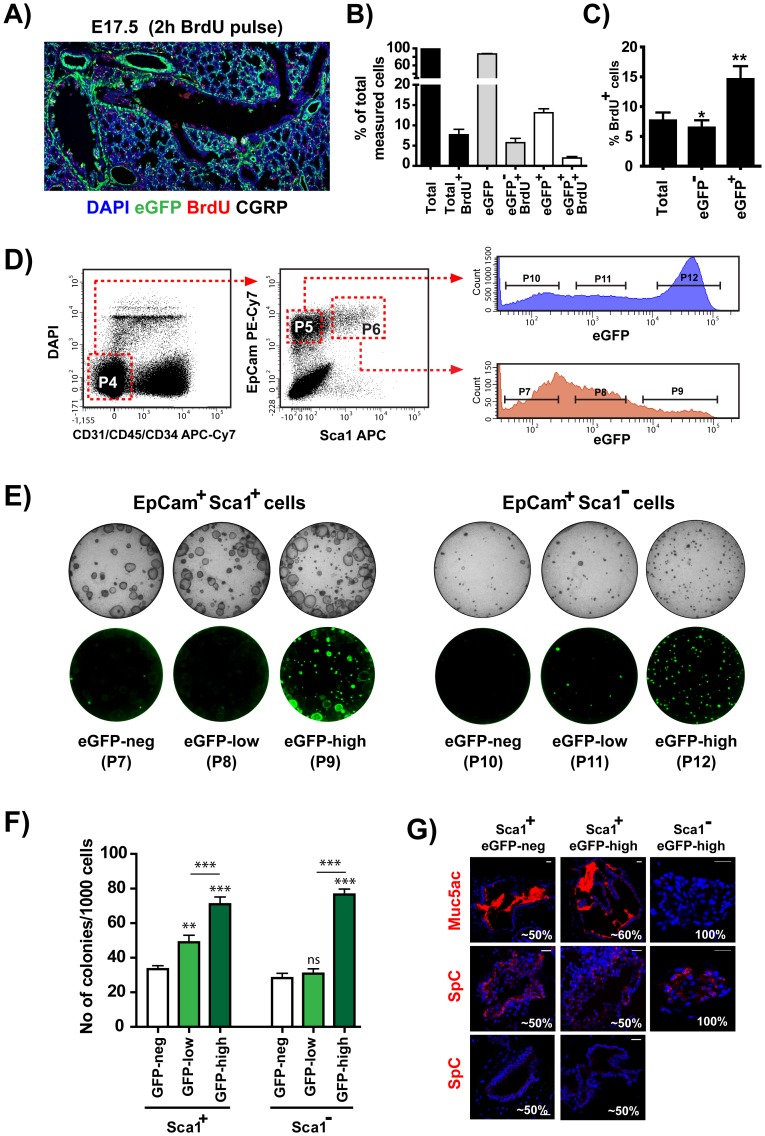
The eGFP^pos^ epithelial compartments are enriched for in-vitro colony-forming epithelial progenitors. A) Confocal image of an E17 lung tissue-section collected after a two-hour pulse with BrdU, stained for eGFP, BrdU, CGRP and DAPI. Colors are as labeled in the figures. Proliferating BrdU^pos^ cells can be seen scattered all across the respiratory tree. B) Percentage of cells with the indicated characteristics in 8000–9000 airway epithelial cells counted per animal. C) Percentage of proliferating cells within each of the indicated epithelial population. Values represent mean ± SEM of six individually-analyzed animals. eGFP^pos^ and eGFP^neg^ groups compared to the “total” group using one-way analysis of variance (ANOVA) with Bonferoni's post-hoc test. D) Isolation of epithelial cell sub-populations by cell sorting. Single cell populations were prepared for cell sorting as described in Materials and Methods. Viable Lin^neg^ cells (gate P4) were subdivided into EpCAM^pos^-Sca1^neg^ (gate P5) and EpCAM^pos^-Sca1^low^ (gate P6). EGFP^neg^, eGFP^low^ and eGFP^high^ cells from either EpCAM^pos^ sub-population were isolated using a FACS ARIA IIu cells sorter. The purities of the sorted sub-populations were: for Sca1^neg^: eGFP^neg^ (gate P7, ∼90%), eGFP^low^ (gate P8, ∼85%), eGFP^high^ (gate P9, ∼90%); for Sca1^low^: eGFP^neg^ (gate P10, ∼95%), eGFP^low^ (gate P11, ∼92%), eGFP^high^ (gate P12, ∼98%). E) Representative bright field and fluorescence (eGFP) images of Day 8 Matrigel cultures seeded with 2000 cells sorted with the gates P7–P12 and co-cultured with Mlg stroma cells in the presence of 10 µM SB431542. Note the dominance of large cystic colonies in the Sca1^low^ sub-populations and the dominance of smaller compact colonies in the Sca1^neg^ sub-populations. After 8 days in culture the vast majority of the colonies from the eGFP^low^ cultured cells were eGFP^neg^. Almost half of the colonies from the eGFP^high^ cultured cells were eGFP^neg^. The frequency of eGFP^pos^ cells in the eGFP^pos^ colonies ranged from 10–100%. F) Quantitation of the colonies produced in Matrigel by 2000 cells from each indicated sorted sub-population.Values represent mean ± SEM of eight independent experiments compared withone-way analysis of variance (ANOVA) with Bonferoni's post-hoc test. **P<0.01, ***P<0.001 (compared to the GFP^neg^ group) and ^###^P<0.001 (eGFP^low^ *vs* eGFP^high^ group). G) SB431542 was removed on day 8 and the Matrigel cultures were incubated for additional four days. Sections of the Matrigel cultures, prepared as described in Materials and Methods, were stained with anti-Muc5Ac and anti-SpC antibodies. All compact colonies derived from the Sca1^neg^ sorted cells were SpC^pos^-Muc5Ac^neg^. The cystic colonies derived from the Sca1^low^ cells after the additional four days culture in the absence of SBSB431542contained Muc5Ac (∼50%) and SpC (∼50%) positive colonies. No Muc5Ac positive colonies were found in cultures with similar cells analyzed on day 8 before the removal of the inhibitor.

The localization of eGFP^pos^ cells in the vicinity of NEBs and the minimal overlap between cells expressing eGFP and markers for specialized secretory (CC10) or ciliated cells (FoxJ1) prompted us to investigate whether active canonical BMP signaling may define cells with an immature progenitor phenotype. Thus, a single two hour pulse of BrdU was given to pregnant mothers at E17 to label cycling epithelial cells which were subsequently detected by staining with anti-BrdU antibodies. BrdU^pos^ cells accounted for ∼6.7% of the surface airway epithelium in conducting airways and were uniformly distributed throughout the airway tree (Figure 7A). This pattern is compatible with the findings of Giangreco et al. [56] and Rawlins et al. [57] demonstrating that randomly distributed progenitor cells contribute to normal epithelial homeostasis. Of the BrdU^pos^ cells ∼77.8% were eGFP^neg^ and ∼22.2% were eGFP^pos^, indicating that both populations contribute to the expansion of the airway epithelial compartment (Figure 7B). Despite the dominance of the eGFP^neg^-BrdU^pos^ cells (in absolute numbers), ∼13.4% of the eGFP^pos^ were BrdU^pos,^ compared to ∼5.5% for the eGFP^neg^ cells (Figure 7C).

To further compare the proliferative potential of the eGFP^pos^ and eGFP^neg^ cells different epithelial populations were isolated from dissociated lung tissue by cell sorting and analyzed*in-vitro* for colony-forming ability. Following a previously described protocol [38], EpCAM^pos^, Lineage negative (i.e. CD45^neg^-CD31^neg^-CD34^neg^) cells from BRE-eGFP transgenic animals were subdivided into Sca1^neg^ and Sca1^low^ cells. Finally the Sca1^neg^ and Sca1^low^ cells were separated by sorting into eGFP^neg^, eGFP^low^ and eGFP^high^ cells respectively (Figure 7D). Isolated cells were co-cultured with MLg feeder cells in a 3-dimensional Matrigel culture system [38]with a modification developed by some of us (HC and BRS) that involved co-culture of the cells in Matrigel for eight days in the presence of the TGFβ receptor inhibitor SB431542 (10 µM), followed by an additional four days in the absence of inhibitor.

Lin^neg^-EpCAM^pos^-Sca1^low^cells cultured in this way, in agreement with previous reports [58], gave rise primarily to large cystic epithelial colonies and Lin^neg^-EpCAM^pos^-Sca1^neg^ cells gave rise almost exclusively to smaller compact epithelial colonies (Figure 7E). The clonogenic capacity of both Sca1^low^ and Sca1^neg^ cells correlated with the expression levels of the BRE-eGFP reporter with the corresponding eGFP^high^ populations exhibiting highest clonogenic capacity (Figure 7F). Previous studies have suggested that cystic colonies derived from Lin^neg^-EpCAM^pos^-Sca1^low^ cells contain progenitors of airway epithelial cells (Muc5Ac^pos^), whereas, the compact colonies derived from Lin^neg^-EpCAM^pos^-Sca1^neg^ cells contain primarily SpC^pos^ alveolar progenitors [58]. Consistently, as shown in Figure 7G, whereas all compact colonies derived from the Sca1^neg^ subpopulations exhibited a similar SpC^pos^-Muc5Ac^neg^ staining pattern, the large cystic colonies derived from the Sca1^low^ subpopulations exhibited a more heterogeneous pattern of staining with approximately half of them expressing Muc5Ac and half of them expressing SpC.

It is worth noting that adult Lin^neg^-EpCAM^pos^-Sca1^neg^ epithelial cells have very low clonogenic capacity in Matrigel [38]. The development of colonies fromLin^neg^-EpCAM^pos^-Sca1^neg^cells in the current study is most probably due to the fact that the analysis was done with P3 lungs which were still undergoing intense alveolar development.

Previous studies have provided evidence for two types of airway epithelial progenitors, one randomly distributed in the airways that maintains normal epithelial homeostasis and responds to minor injuries of the epithelium, and a second, associated with previously described progenitor cell niches that can renew depleted Clara cells upon severe epithelial injury [38], [56]. The randomly distributed eGFP^neg^-BrdU^pos^ cells detected in the developing lungs (Figure 7A) could represent the former type whereas the eGFP^pos^ epithelial cells could represent the latter type of progenitors. Once lung development is completed, the homeostatic, low turnover, maintenance of the epithelial population can be supported by the distributed Clara cells [38], [56] and thus active BMP signaling may not be required any longer. The decline in eGFP expression in the lungs of the adult BRE-eGFP transgenics may reflect just that.

### Reactivation of the BRE-eGFP reporter in adult lung after tissue injury suggests an active role of BMP-signaling in adult lung repair after injury

**Figure 8:**
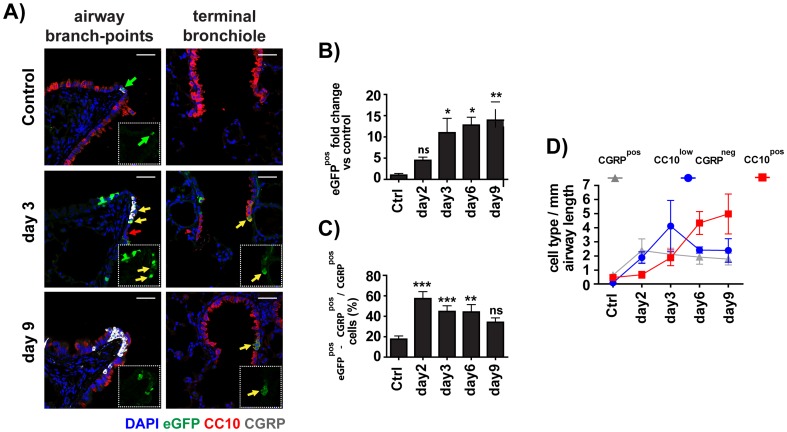
The eGFP reporter is reactivated in the airways of BRE-eGFP transgenic animals after injury of their bronchial epithelium by naphthalene. A) Confocal images of lung tissues collected from vehicle (corn oil) or naphthalene-treated BRE-eGFP transgenic animals, 3 and 9 days post-treatment, stained for eGFP, CC10 and CGRP. Colors are as labeled in the figure. Nuclei were counterstained with DAPI. Reactivation of the eGFP reporter is shown in the vicinity of NEBs or terminal-bronchiolar regions. Scale: 40 µm. B) Morphometric analysis of eGFP^pos^ cells in naphthalene-treated animals expressed as fold increase over the vehicle-treated controls. C) Morphometric analysis illustrating the increased frequency of eGFP expression among the CGRP^pos^ cells. Values represent mean ± SEM of 4–5 individually analyzed naphthalene-treated animals per group compared to the vehicle control group (n = 7) using one-way analysis of variance (ANOVA) with Bonferoni's post-hoc test. *P<0.05, **P<0.01, and ***P<0.001. D) Morphometric kinetic analysis demonstrating the relative numbers of CGRP^pos^, CC10^pos^, and CC10^low^ cells among the naphthalene-induced eGFP^pos^ airway-epithelial cells, expressed as average number of cells per mm airway length. Values represent mean ± SEM of 4–5 individually analyzed naphthalene-treated animals per group at each time point. Note the sequential increase of eGFP^pos^-CGRP^pos^, eGFP^pos^-CC10^low^and eGFP^pos^-CC10^pos^ cells.

The dramatic decline of eGFP expression in the adult lung of the BRE-eGFP reporter mice and the association of eGFP expression with the epithelial progenitor pool prompted us to investigate whether the reporter was reactivated in adult lung during injury and repair. Adult BRE-eGFP animals were analysed in two established models of lung tissue injury and repair, namely the naphthalene and bleomycin induce lung injury models. Naphthalene treatment causes selective depletion of Clara cells in the airways [59]. In contrast, bleomycin causes epithelial cell death, acute inflammation and fibro-proliferative remodeling of the peripheral lung [60]. Immuno-staining for CC10 and CGRP demonstrated that by day 3, naphthalene treatment had caused a large reduction in CC10^pos^ cells in the airways, an increase in the number of CGRP^pos^ cells per NEB, and a substantial increase in BRE-eGFP reporter activity at airway branch-points and terminal bronchioles (Figure 8A).This pattern of CC10 localization and eGFP reporter expression corresponds with previously determined anatomic locations that maintain chemically resistant epithelial progenitor cells [61], [62], [63], [64].

Analysis at different time points after naphthalene-treatment demonstrated a gradual increase in the number of eGFP^pos^ bronchial epithelial cells that was evident on day 2 and maximum increase of 15-fold over control levels by day-9 post-naphthalene treatment (Figure 8B). Up to day-2, the increase in eGFP^pos^ airway epithelial cell numbers involved CGRP^pos^ and CGRP^neg^-CC10^low^cells (Figures 8C and D). Thereafter, although the total number of eGFP^pos^-CGRP^pos^ cells remained stable from days 2–9, a wave of eGFP^pos^-CC10^low^-CGRP^neg^ cells that reached maximum around day 3 was followed by a wave of eGFP^pos^-CC10^pos^ cells that reached plateau around day-9 (Figure 8D).

Interestingly, the distribution of eGFP and CC10 expression recapitulated the pattern observed during early lung development i.e. eGFP^pos^-CC10^pos^ cells separating eGFP^pos^-CC10^low^ from apparently newly formed eGFP^neg^-CC10^pos^ cells. The kinetics of appearance and relative localization of these cells within the tissues are consistent with the notion that they may represent sequential stages of development from the eGFP^pos^-CC10^low^ to the eGFP^neg^-CC10^pos^ phenotype.

**Figure 9:**
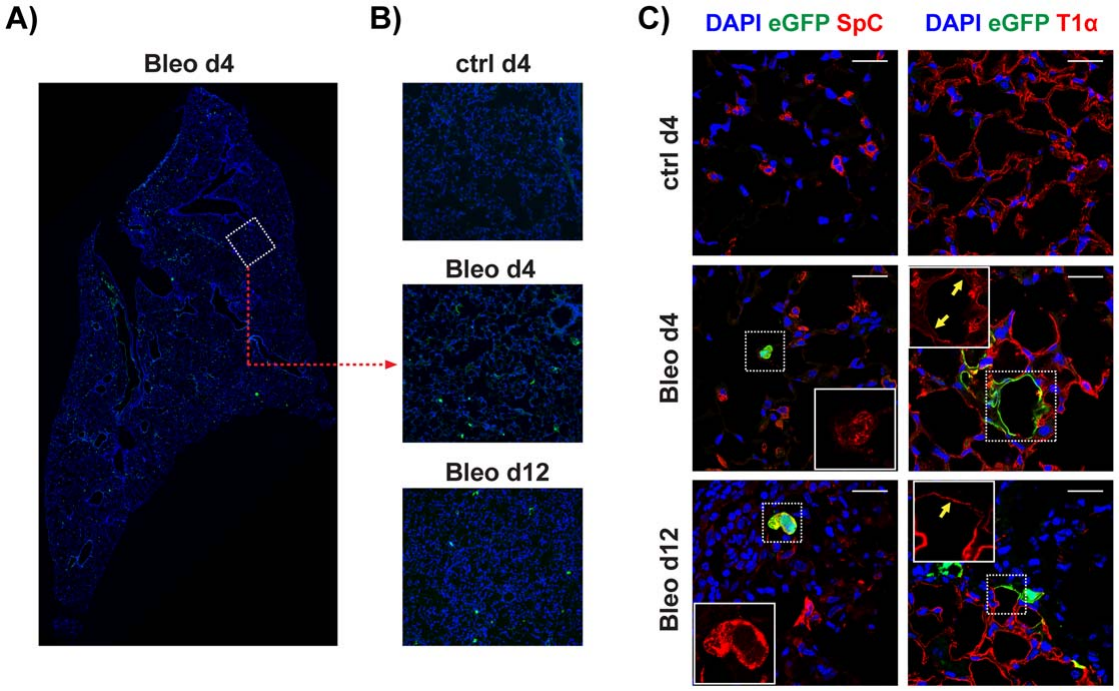
The eGFP reporter is reactivated in the alveolar regions of BRE-eGFP transgenic animals after bleomycin treatment. **A**) Panoramic picture produced by digitally merging fifty-four 10× images from a lung tissue-section of a BRE-eGFP animal analysed four days after intra-tracheal instillation of bleomycin. Numerous eGFP^pos^ cells are scattered in alveolar regions. B) Representative higher magnification images of BRE-eGFP transgenics treated with vehicle or bleomycin(days 4 and 12 post-instillation) demonstrating the appearance of eGFP^pos^ alveolar-cells (scale bar: 150 µm. C) Representative confocal images of tissue sections stained for eGFP, SpC and T1α, demonstrating, eGFP expression in SpC^pos^ and T1α^low^cells. Scale bar: 25 µm.

Bleomycin is an anti-cancer drug, which induces lung injury and fibrosis [60]. As shown in Figures 9A and 9B, treatment of adult BRE-eGFP animals by intra-tracheal administration of bleomycin resulted in substantial reactivation of the eGFP reporter in type-II pneumocytes (Pro-SpC^pos^). During the first days of treatment the eGFP^pos^ cells were primarily cuboidal SpC^pos^ cells. From day-4 onwards both cuboidal and squamous eGFP^pos^ cells were found in the alveolar regions. Interestingly, the squamous eGFP^pos^ cells were T1α-expressing type-I pneumocytes and were always localized in the borders between affected and apparently normal tissue (Figure 9C). We postulate that the cuboidal eGFP^pos^ cells observed in the early stages of the bleomycin induced response represent a reparatory progenitor population that expands and differentiates into the eGFP^pos^-T1α^low^ cells observed in the later stages to repair the damaged alveolar epithelium. Further studies will be required, however, to validate this hypothesis.

Collectively, our findings demonstrate that the canonical BMP pathway is re-activated during adult lung injury in a manner that bears resemblance to the activation of this pathway during early lung development, strongly supporting its importance during adult lung tissue injury repair.

## Conclusions

Utilizing a transgenic reporter mouse line harboring a BMP-responsive eGFP reporter allele we were able to construct a detailed spatiotemporal map of canonical BMP signalling during early lung development and adult lung tissue injury repair. Our studies demonstrated that during the pseudoglandular stage, when branching morphogenesis characterises lung development, canonical BMP pathway is active mainly in the vascular network and the airway smooth muscle layer. Only after the completion of branching morphogenesis and the initiation of epithelial cell differentiation canonical BMP pathway activation commences in airway and alveolar epithelial cell that are enriched in progenitors that can form epithelial colonies in Matrigel *in-vitro*. The BRE-eGFP reporter is reactivated in mesenchyme-free distal epithelial buds cultured in Matrigel suggesting that the low reporter activity in intact lungs is the result of a dynamic interplay between the endoderm and the mesenchyme and moreover suggest that the elusive, mesenchyme derived, epithelial growth promoting signal postulated by Bragg et al. [51] may act by regulating negatively activation of the canonical BMP pathway in the distal epithelium buds.

BRE-eGFP expression, in agreement with earlier reports describing the temporal expression pattern of some BMP pathway components during late lung development [65], peaks around birth and returns to very low levels upon completion of lung development. Remarkably, severe depletion of Clara cells in the adult lung by naphthalene treatment, leads to re-expression of the eGFP reporter around NEBs and terminal bronchioles, areas known to harbour airway progenitor cells. Likewise, injury of the alveolar epithelium in the adult lung by bleomycin treatment, leads to re-expression of eGFP initially in SpC^pos^ cuboidal epithelial cells, which we postulated to be alveolar epithelial progenitors and subsequently in squamous T1α^low^ cells which we postulated to be derived from the former population and on the way to differentiate to Type-I pneumonocytes. Our findings are compatible with the notion that during the late stages of lung development the role of canonical BMP signalling pathway is to maintain the undifferentiated state of the airway and alveolar epithelial progenitors preventing premature exhaustion of their pools and securing the continuous supply of the differentiated epithelial cells that are required for a fully developed lung. The reactivation of the BMP responsive reporter in adult animals undergoing repair of severe epithelial injury, where the same requirements regarding proper management of the undifferentiated epithelial progenitor pools apply, is also compatible with this conjecture. An analogous function has been previously proposed for BMP signaling regarding the maintenance of the undifferentiated state of pluripotent mouse embryonic stem (ES) cells [66], [67] and the maintenance of the undifferentiated, multipotent state of distal lung progenitor cells [45], [68].

**Figure 10:**
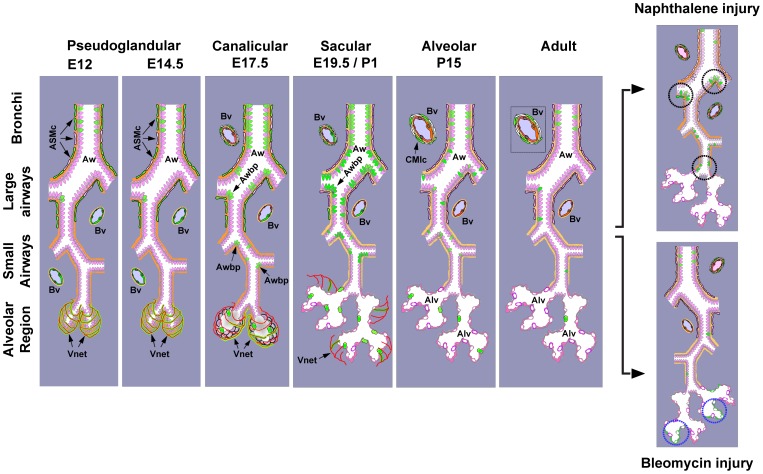
Schematic representation of the spatiotemporal activation pattern of Canonical BMP pathway in the mouse lung. Summary of canonical BMP-signaling activation in lung development and adult tissue-repair based on the current study and the studies of Monteiro et al. [33] and Blank et al. [39]. Airways (Aw), airway branch-points (Awbp), blood-vessel (Bv), Airway smooth muscle cells (ASMc), cardiomyocyte-like cells (CMlc), alveolar regions (Alv). eGFP^pos^ cells are shown green.

The construction of a spatiotemporal map of canonical BMP signalling during lung development and adult lung tissue injury repair (summarised in Figure 10 and table S2) will facilitate interpretation of earlier genetic studies and guide rational targeting of BMP signaling components in future experiments. Moreover, the ease isolation of living eGFP^pos^ i.e. BMP responding, subpopulations of lung cells by cell sorting will greatly facilitate definitive clarification of the mechanisms of action of this signaling system during early lung development and repair of lung tissue injury in the adult.

## Supporting Information

Figure S1 **Activation of canonical BMP-pathway in E12.5 lungs.** A) Representative confocal images demonstrating activation of the BRE-eGFP transgene in the vascular network of the lung in E12 embryos. E12 lung tissues from BRE-eGFP transgenic animals were stained for eGFP, CD31, VEGFR-2 and αSMA. Nuclei were counterstained with DAPI. The images demonstrate robust activation of eGFP expression in CD31^pos^ and VEGFR-2^pos^ cells and week activation in some of the sub-epithelial αSMA^pos^ cells (see insert in lower right image). Of note is the complete absence of any eGFP staining in the airway epithelial compartment. B) Kinetics of BRE-eGFP reporter activation in sub-epithelial smooth muscle cells (CMSs). Representative confocal images of tissue sections prepared from E11, E12, E13.5 and E14.5 BRE-eGFP transgenic lungs and stained for eGFP, SM22a and αSMA. The left panel shows magnified the eGFP channel only. The white dashed rectangles in each lane of images correspond to identical tissue areas. Note that the BRE-eGFP reporter is activated in mature aSMA positive cells that are located in the most proximal portion of the airway tree.
(TIF)

Figure S2 **Activation of the canonical BMP-pathway in sub-epithelial smooth muscle cells of the proximal airways in E14.5 lungs.** Representative confocal image of an E14.5 tissue section stained for eGFP (green staining) and SpC (red staining). Nuclei were counterstained with DAPI. Only proximal airways (defined by the very low staining for Pro-SpCare surrounded by eGFP^pos^ sub-epithelial smooth muscle cells.
(TIF)

Figure S3 **FGF10, BMP4 and a Sonic Hedgehog agonist affect expression of the BRE-eGFP reporter.** A) Whole E12 lung-explants were cultured on Nuclepore membranes for eight hours in the presence of vehicle, FGF10 (250 ng/ml), BMP4 (100 ng/ml) or Sonic Hedgehog agonist Purmorphamin (500 nM). Upper panel shows bright field images of representative explants. Lower panel shows eGFP expression in the same explants. B) Confocal images of tissue sections prepared from the explants described above stained for eGFP (green stain). Nuclei were counterstained with DAPI. Note the strong upregulation of eGFP expression in the FGF10 and BMP4 treated explants, and the contruction of the eGFP positive zone in the purmorphamine treated group.
(TIF)

Figure S4 **Correlation between pSmad1/5/8 and BRE-eGFP immune-staining in P1 lung tissue sections.** Adjacent tissue section of lungs from BRE-eGFP reporter animals were stained with anti-GFP or anti-pSmad1/5/8 antibodies as described in materials and methods. A) Staining of adjacent sections of a large airway with anti-GFP and anti pSmad1/5/8antibodies or normal rabbit IgG fraction as isotype control. B) Representative images from the indicated tissue regions demonstrating that tissue areas with intense BRE-eGFP expression coincide with regions exhibiting pSma1/5/8 immuno-staining. The scale bar corresponds to 25 µm.
(TIF)

Figure S5 **The zone of eGFP^pos^CC10^pos^ cells coincides with the NEB-rich portion of the airway tree.** A) Confocal image of a lung tissue section derived from a P1 BRE-eGFP animal stained for eGFP (green staining), CC10 (red staining) and CGRP (white staining). The images illustrate the zone in the airway-tree where co-expression of eGFP and CC10 occurs. The lower images, showing the CGRP and DAPI channels of the upper images, illustrate the coincidence of eGFP^pos^-CC10^pos^ zone with the NEB-rich regions of the airway tree. B) Confocal images of the transitional zone between the eGFP^pos^-CC10^neg^ and eGFP^neg^-CC10pos domains of the airways demonstrating the preferential association of eGFP^pos^-CC10^low^ cells with NEBs. The image depicting NEBs is obtained from a section sequential to the ones depicting CC10 and eGFP expression. Nuclei were counterstained with DAPI (blue staining).
(TIF)

Figure S6 **Minimal eGFP expression in the Foxj-1^pos^ airway ciliated cells.** Representative confocal images demonstrating minimal activation of the BRE-eGFP transgene in FoxJ1^pos^ ciliated airway epithelial cells. Tissue section collected from E17.5, E19.5, P1, P15 and adult BRE-eGFP transgenic lungs were stained for eGFP (green staining), FoxJ1 (red staining) and CGRP (white staining). Nuclei were counterstained with DAPI (blue staining). The images demonstrate the presence of remarkably low number of eGFP^pos^-FoxJ1^pos^ epithelial cell (depicted with yellow arrows).
(TIF)

Figure S7 **A small, however, detectable number of BRE-eGFP developing type-I pneumocytes (T1**α**^pos^) express eGFP.** Representative confocal images of lung sections of E14.5, E17.5, E19.5, P1, P15 and adult BRE-eGFP transgenic lungs stained for eGFP (green staining), CD31 (red staining) and T1α (white staining). Nuclei were counterstained with DAPI (blue staining). The Scale bars are 20 µm. The images demonstrate that whereas the majority of the developing endothelial cells express eGFP, only a small number of type-I pneumocytes, shown in the inserts of the P1 and P15 image, are eGFP^pos^.
(TIF)

Table S1 **Primer pairs utilized for quantitative PCR analysis in the present study.** The forward and reverse primers designed with the Beacon Designer v7.01 software, using the indicated data-base gene sequences (Ref. sequence) and the size of the amplicons are indicated.
(DOCX)

Table S2 Summary of the spatiotemporal expression pattern of the BRE-eGFP reporter during lung development and adult lung tissue injury repair.
(DOCX)
