# Supplementary figures and images for: Activation of the Canonical Bone Morphogenetic Protein (BMP) Pathway during Lung Morphogenesis and Adult Lung Tissue Repair
Source: PLoS One. 2012 Aug 20;7(8):e41460. doi: 10.1371/journal.pone.0041460 (PMC3423416; doi:10.1371/journal.pone.0041460)

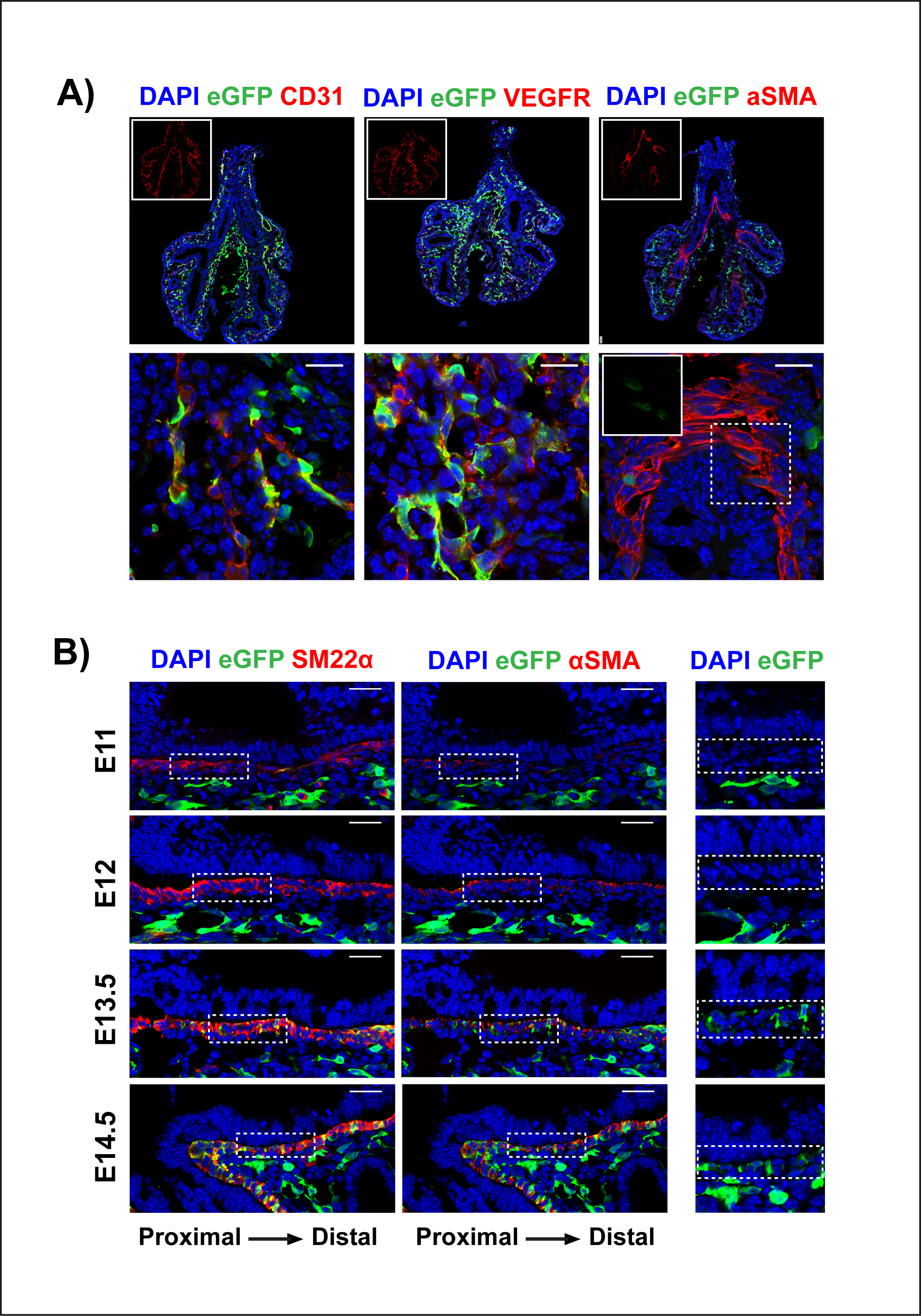

Supplement: Figure S1 — Activation of canonical BMP-pathway in E12.5 lungs. A) Representative confocal images demonstrating activation of the BRE-eGFP transgene in the vascular network of the lung in E12 embryos. E12 lung tissues from BRE-eGFP transgenic animals were stained for eGFP, CD31, VEGFR-2 and αSMA. Nuclei were counterstained with DAPI. The images demonstrate robust activation of eGFP expression in CD31pos and VEGFR-2pos cells and week activation in some of the sub-epithelial αSMApos cells (see insert in lower right image). Of note is the complete absence of any eGFP staining in the airway epithelial compartment. B) Kinetics of BRE-eGFP reporter activation in sub-epithelial smooth muscle cells (CMSs). Representative confocal images of tissue sections prepared from E11, E12, E13.5 and E14.5 BRE-eGFP transgenic lungs and stained for eGFP, SM22a and αSMA. The left panel shows magnified the eGFP channel only. The white dashed rectangles in each lane of images correspond to identical tissue areas. Note that the BRE-eGFP reporter is activated in mature aSMA positive cells that are located in the most proximal portion of the airway tree. (TIF) [file pone.0041460.s001.tif]

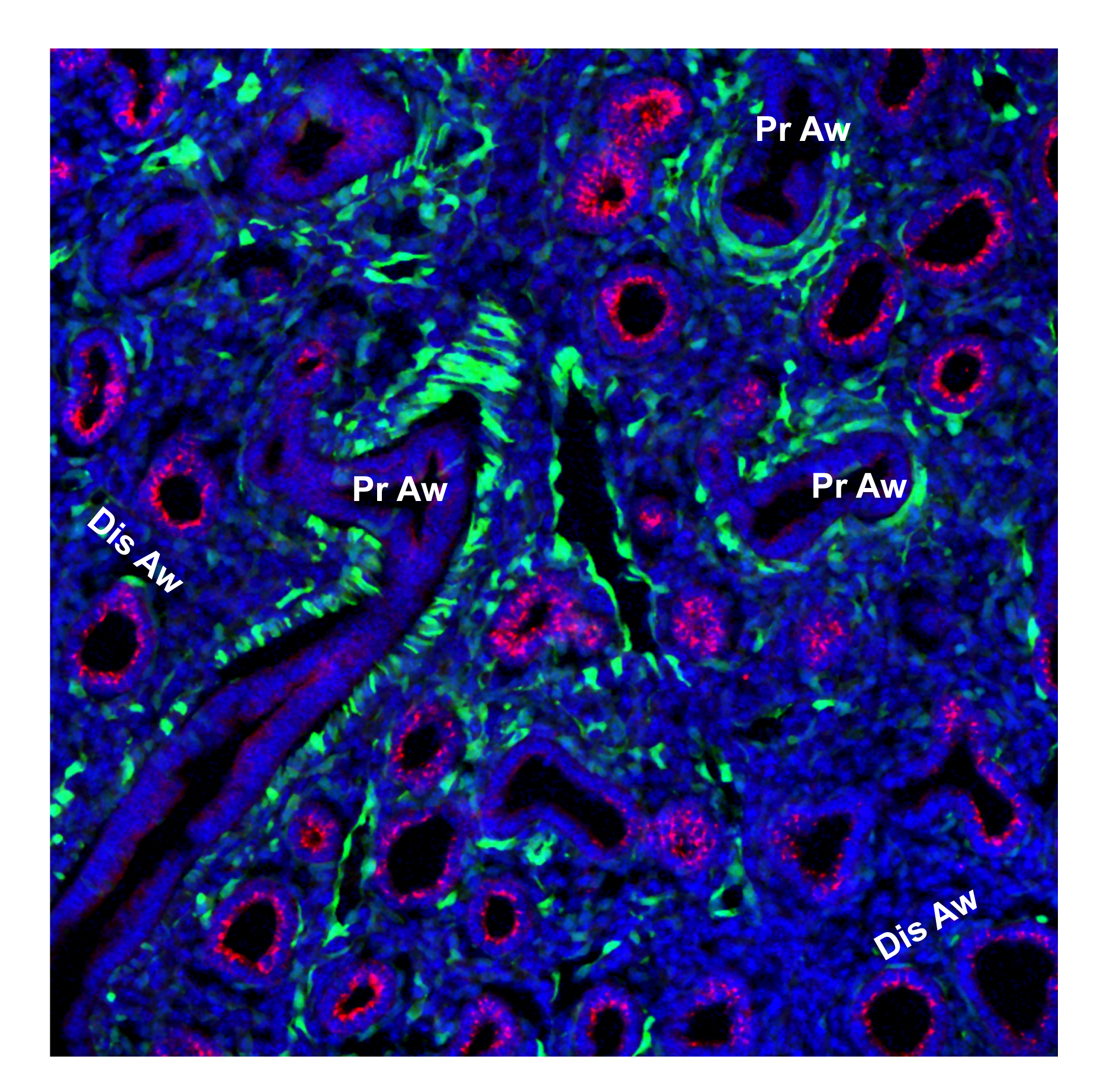

Supplement: Figure S2 — Activation of the canonical BMP-pathway in sub-epithelial smooth muscle cells of the proximal airways in E14.5 lungs. Representative confocal image of an E14.5 tissue section stained for eGFP (green staining) and SpC (red staining). Nuclei were counterstained with DAPI. Only proximal airways (defined by the very low staining for Pro-SpCare surrounded by eGFPpos sub-epithelial smooth muscle cells. (TIF) [file pone.0041460.s002.tif]

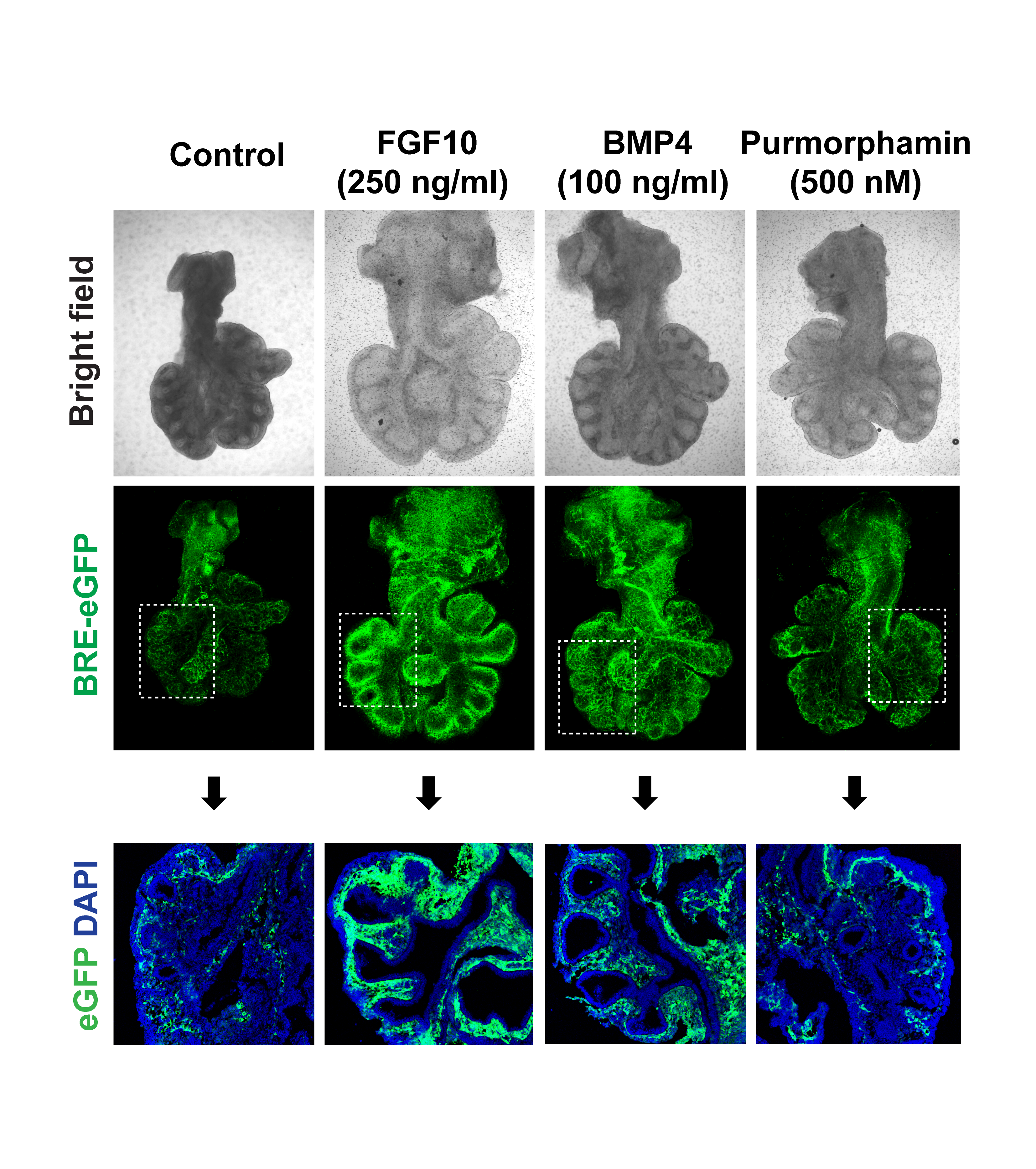

Supplement: Figure S3 — FGF10, BMP4 and a Sonic Hedgehog agonist affect expression of the BRE-eGFP reporter. A) Whole E12 lung-explants were cultured on Nuclepore membranes for eight hours in the presence of vehicle, FGF10 (250 ng/ml), BMP4 (100 ng/ml) or Sonic Hedgehog agonist Purmorphamin (500 nM). Upper panel shows bright field images of representative explants. Lower panel shows eGFP expression in the same explants. B) Confocal images of tissue sections prepared from the explants described above stained for eGFP (green stain). Nuclei were counterstained with DAPI. Note the strong upregulation of eGFP expression in the FGF10 and BMP4 treated explants, and the contruction of the eGFP positive zone in the purmorphamine treated group. (TIF) [file pone.0041460.s003.tif]

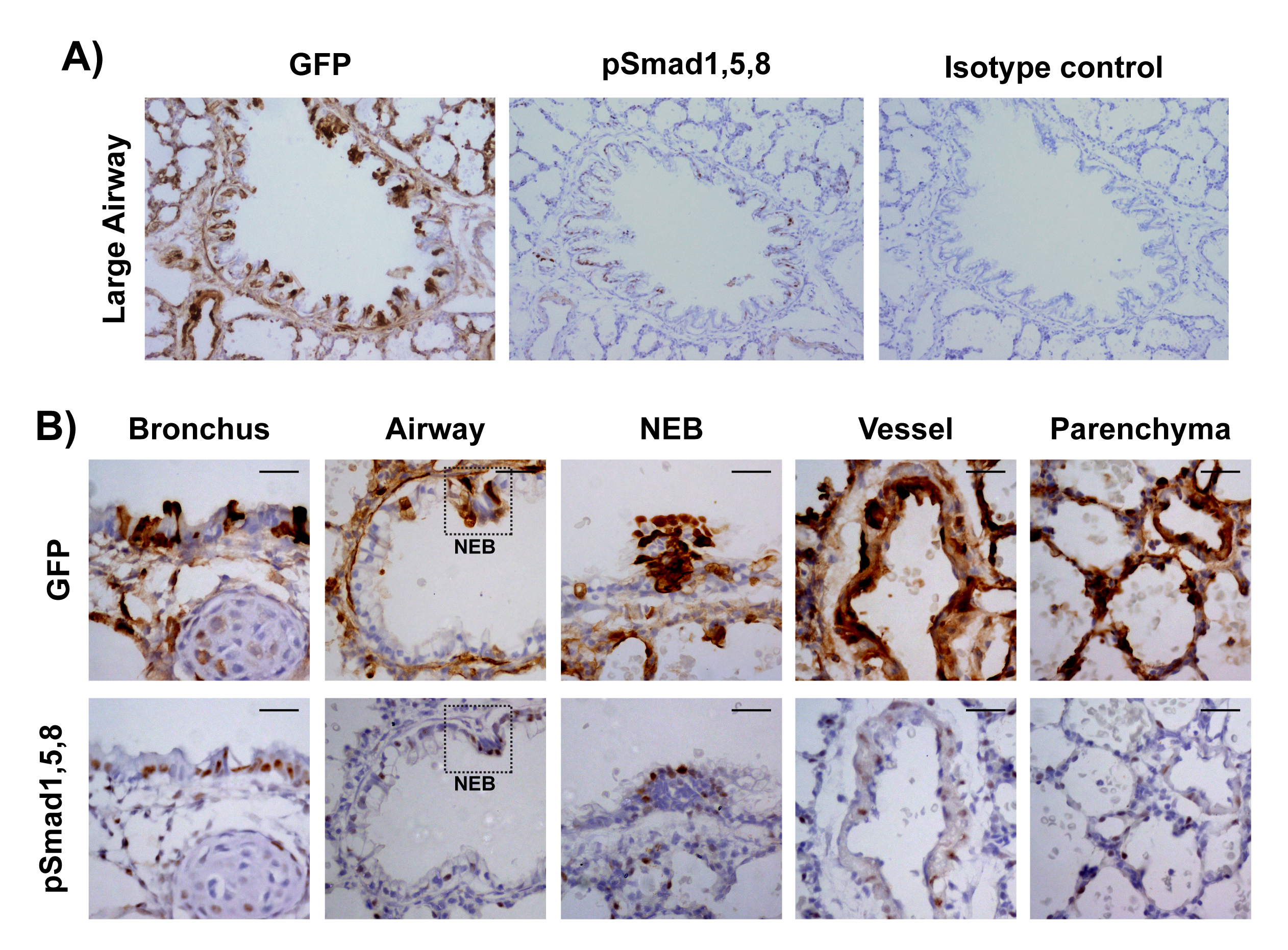

Supplement: Figure S4 — Correlation between pSmad1/5/8 and BRE-eGFP immune-staining in P1 lung tissue sections. Adjacent tissue section of lungs from BRE-eGFP reporter animals were stained with anti-GFP or anti-pSmad1/5/8 antibodies as described in materials and methods. A) Staining of adjacent sections of a large airway with anti-GFP and anti pSmad1/5/8antibodies or normal rabbit IgG fraction as isotype control. B) Representative images from the indicated tissue regions demonstrating that tissue areas with intense BRE-eGFP expression coincide with regions exhibiting pSma1/5/8 immuno-staining. The scale bar corresponds to 25 µm. (TIF) [file pone.0041460.s004.tif]

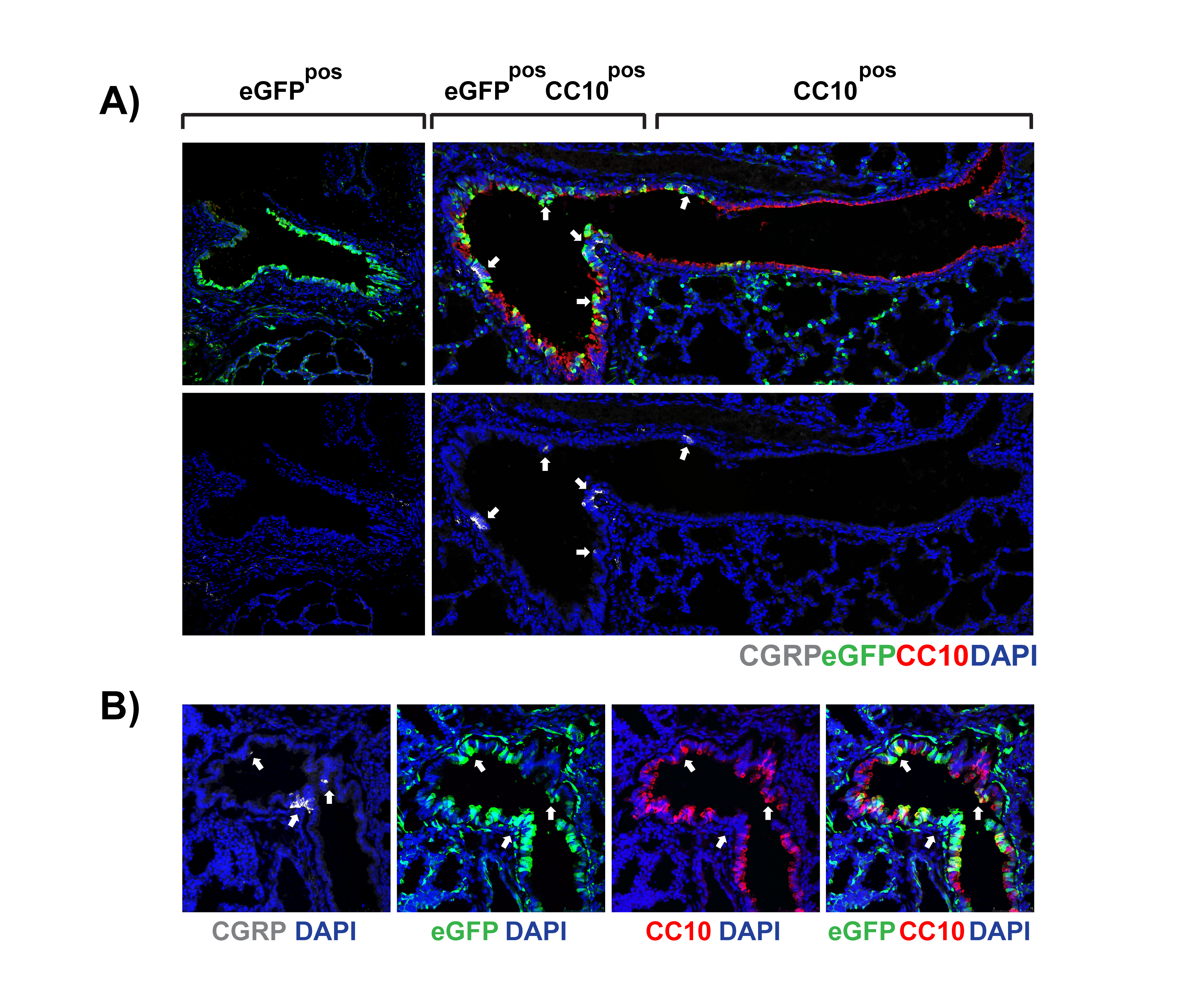

Supplement: Figure S5 — The zone of eGFPposCC10pos cells coincides with the NEB-rich portion of the airway tree. A) Confocal image of a lung tissue section derived from a P1 BRE-eGFP animal stained for eGFP (green staining), CC10 (red staining) and CGRP (white staining). The images illustrate the zone in the airway-tree where co-expression of eGFP and CC10 occurs. The lower images, showing the CGRP and DAPI channels of the upper images, illustrate the coincidence of eGFPpos-CC10pos zone with the NEB-rich regions of the airway tree. B) Confocal images of the transitional zone between the eGFPpos-CC10neg and eGFPneg-CC10pos domains of the airways demonstrating the preferential association of eGFPpos-CC10low cells with NEBs. The image depicting NEBs is obtained from a section sequential to the ones depicting CC10 and eGFP expression. Nuclei were counterstained with DAPI (blue staining). (TIF) [file pone.0041460.s005.tif]

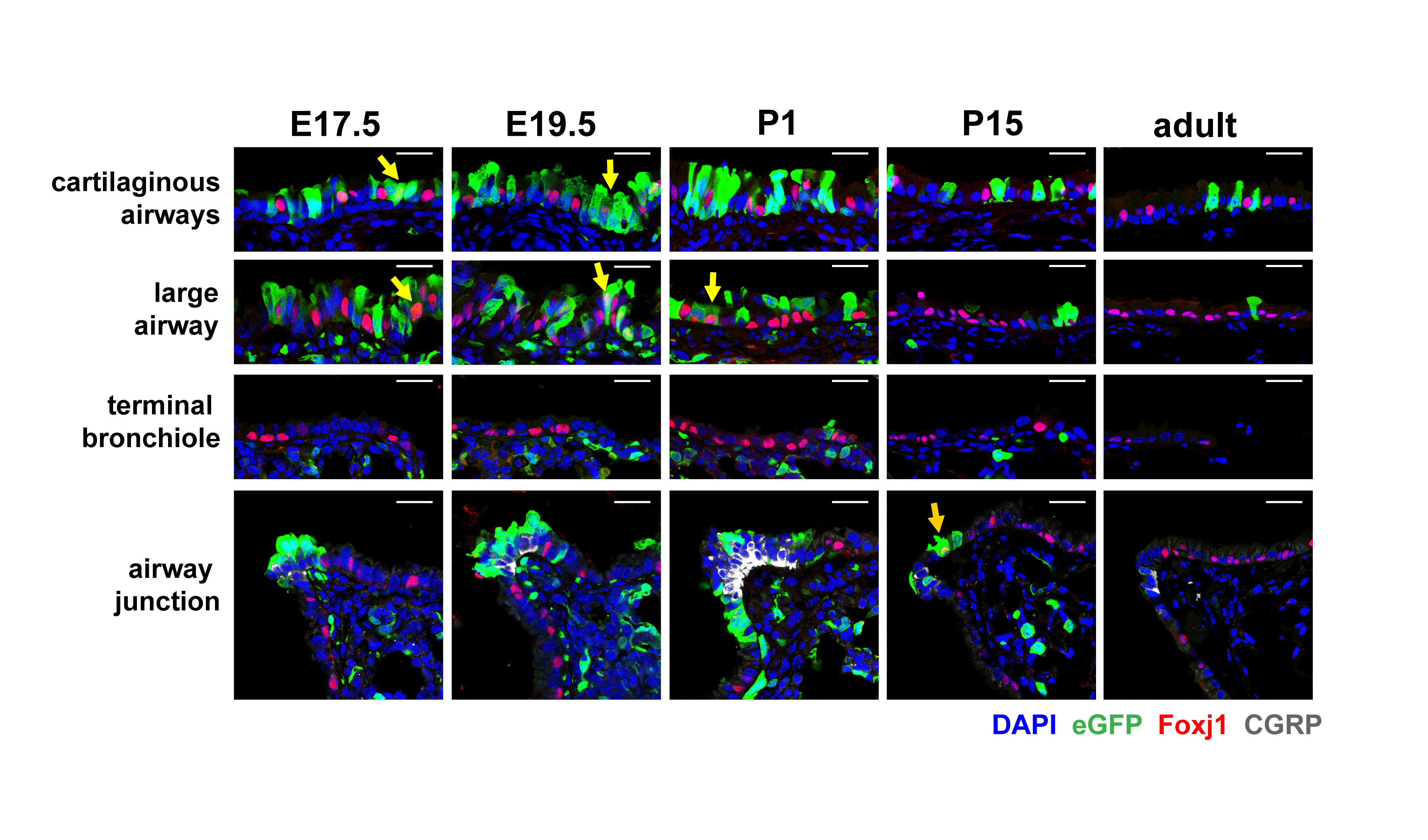

Supplement: Figure S6 — Minimal eGFP expression in the Foxj-1pos airway ciliated cells. Representative confocal images demonstrating minimal activation of the BRE-eGFP transgene in FoxJ1pos ciliated airway epithelial cells. Tissue section collected from E17.5, E19.5, P1, P15 and adult BRE-eGFP transgenic lungs were stained for eGFP (green staining), FoxJ1 (red staining) and CGRP (white staining). Nuclei were counterstained with DAPI (blue staining). The images demonstrate the presence of remarkably low number of eGFPpos-FoxJ1pos epithelial cell (depicted with yellow arrows). (TIF) [file pone.0041460.s006.tif]

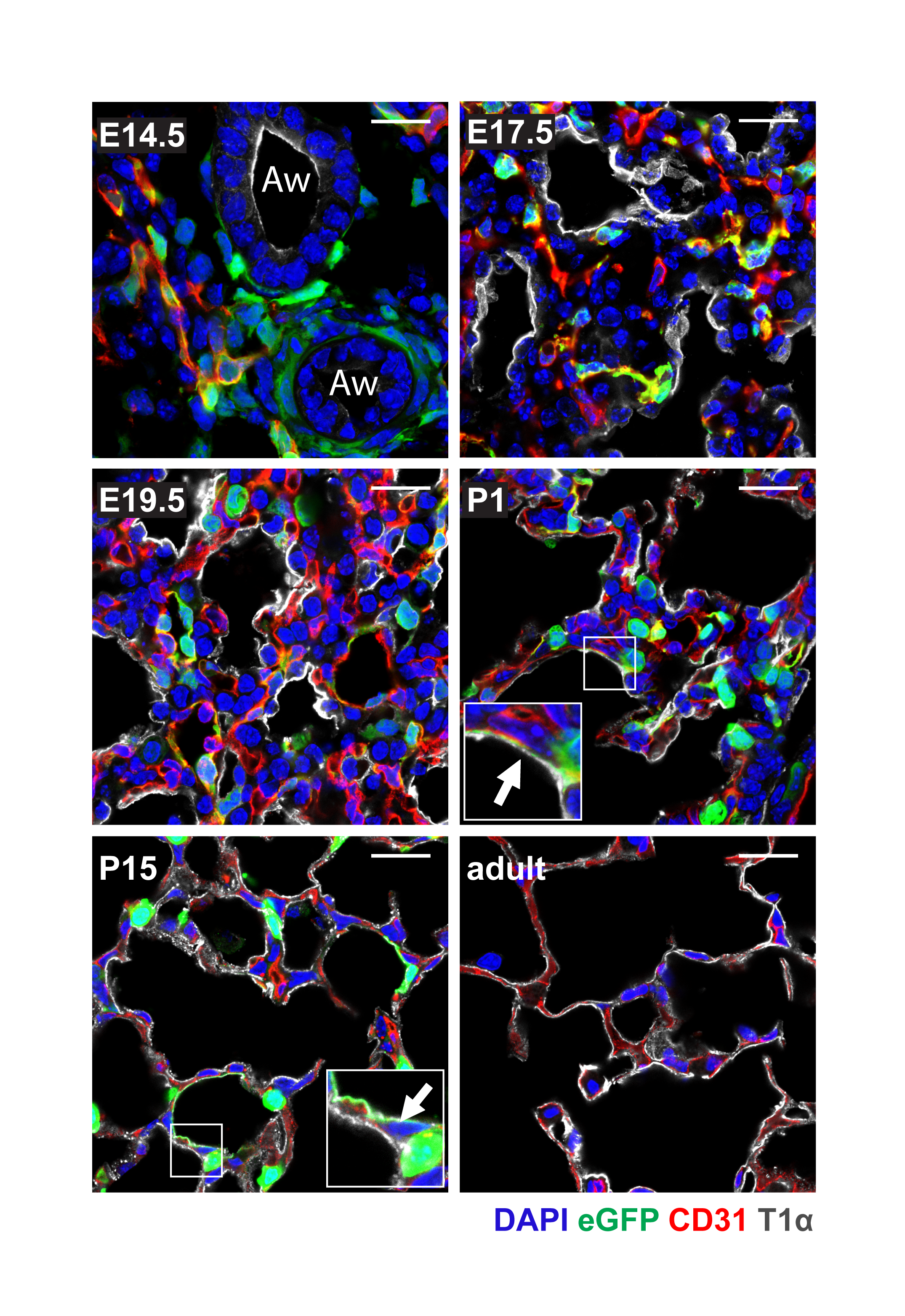

Supplement: Figure S7 — A small, however, detectable number of BRE-eGFP developing type-I pneumocytes (T1αpos) express eGFP. Representative confocal images of lung sections of E14.5, E17.5, E19.5, P1, P15 and adult BRE-eGFP transgenic lungs stained for eGFP (green staining), CD31 (red staining) and T1α (white staining). Nuclei were counterstained with DAPI (blue staining). The Scale bars are 20 µm. The images demonstrate that whereas the majority of the developing endothelial cells express eGFP, only a small number of type-I pneumocytes, shown in the inserts of the P1 and P15 image, are eGFPpos. (TIF) [file pone.0041460.s007.tif]
