# Supplementary material for: Activation of the Canonical Bone Morphogenetic Protein (BMP) Pathway during Lung Morphogenesis and Adult Lung Tissue Repair
Source: PLoS One. 2012 Aug 20;7(8):e41460. doi: 10.1371/journal.pone.0041460 (PMC3423416; doi:10.1371/journal.pone.0041460)
Supplement: Table S1 — Primer pairs utilized for quantitative PCR analysis in the present study. The forward and reverse primers designed with the Beacon Designer v7.01 software, using the indicated data-base gene sequences (Ref. sequence) and the size of the amplicons are indicated. (DOCX) [file pone.0041460.s008.docx]

**Supplemental Table S1.**

| **GENE** | **Ref. sequence** | **Primers** | | **product** |
| --- | --- | --- | --- | --- |
|  |  | **Forward (5’→3’)** | **Reverse (5’→3’)** |  |
| **eGFP** | U55762 | CATCTTCTTCAAGGACGAC | TTGTGGCTGTTGTAGTTG | 152 bp |
| **Id1** | [NM_010495](http://www.ncbi.nlm.nih.gov/entrez/viewer.fcgi?val=NM_010495.2) | GGCGAGATCAGTGCCTTG | AAGGGCTGGAGTCCATCTG | 106 bp |
| **Id2** | [NM_010496](http://www.ncbi.nlm.nih.gov/entrez/viewer.fcgi?val=NM_010496.3) | ATGAAAGCCTTCAGTCCGGTG | AGCAGACTCATCGGGTCGT | 107 bp |
| **Id3** | [NM_008321](http://www.ncbi.nlm.nih.gov/entrez/viewer.fcgi?val=NM_008321.2) | CTTAGCCAGGTGGAAATC | CTTGTCCTTGGAGATCAC | 153 bp |
| **Smad6** | [NM_008542](http://www.ncbi.nlm.nih.gov/entrez/viewer.fcgi?db=nuccore&id=31560672) | AATTCTCAGATGCCAGCATG | AGGTAGGTCGTAGAAGATGC | 140bp |
| **FGF10** | NM_008002 | CGCTGGAGAAGGCTGTTC | CTAAGTAATAGTTGCTGTTGATGG | 166 bp |
| **VEGFα** | [NM_001025250](http://www.ncbi.nlm.nih.gov/entrez/viewer.fcgi?val=NM_001025250.3) | CTGTAACGATGAAGCCCTGGAG | TGGTGAGGTTTGATCCGCAT | 81 bp |
| **BMP4** | [NM_007554](http://www.ncbi.nlm.nih.gov/entrez/viewer.fcgi?db=nuccore&id=121949822) | GAGGAGGAGGAAGAGCAGAG | TCTCCAGATGTTCTTCGTGATG | 106 bp |
| **gapdh** | [NM_008084](http://www.ncbi.nlm.nih.gov/entrez/viewer.fcgi?db=nuccore&id=126012538) | CCAGTATGACTCCACTCACG | CTCCTGGAAGATGGTGATGG | 97 bp |
