# Supplementary material for: Activation of the Canonical Bone Morphogenetic Protein (BMP) Pathway during Lung Morphogenesis and Adult Lung Tissue Repair
Source: PLoS One. 2012 Aug 20;7(8):e41460. doi: 10.1371/journal.pone.0041460 (PMC3423416; doi:10.1371/journal.pone.0041460)
Supplement: Table S2 — Summary of the spatiotemporal expression pattern of the BRE-eGFP reporter during lung development and adult lung tissue injury repair. (DOCX) [file pone.0041460.s009.docx]

**Supplemental Table S2.**

| **Lung**  **compartment** | **E11** | **E12** | **E13.5** | **E14.5** | **E17.5** | **E19.5** | **P1** | **P15** | **Adult** | **Naphthalene**  **injury** | **Bleomycin injury** |
| --- | --- | --- | --- | --- | --- | --- | --- | --- | --- | --- | --- |
| Vascular endothelium | **++** | **++** | **+++** | **+++** | **+++** | **++** | **++** | **-** | **-** | **-** | **-** |
| Airway smooth muscle |  | **+/-** | **++** | **+++** | **+++** | **+++** | **+++** | **+** | **-** | **-** | **-** |
| NEBs |  | **-** |  | **-** | **++** | **+++** | **+++** | **++** | **-** | **++** | **-** |
| Bronchial epithelium |  | **-** |  | **-** | **++** | **+++** | **+++** | **+** | **-** | **+** | **-** |
| Alveolar Epithelium |  | **-** |  | **-** | **+** | **++** | **+++** | **++** | **-** | **-** | **++** |
| Pulmonary cardiomyocytes |  | **-** |  | **-** | **+** | **++** | **++** | **+++** | **+++** | **+++** | **+++** |
